# Platform workers not by chance: Exploring the digital labour markets in Italy with machine learning and explainable AI

**DOI:** 10.1371/journal.pone.0340237

**Published:** 2026-06-30

**Authors:** Clara Punzi, Valeria Cirillo, Dario Guarascio, Roberto Pellungrini, Fosca Giannotti

**Affiliations:** 1 Faculty of Science, Scuola Normale Superiore, Pisa, Italy; 2 Department of Computer Science, University of Pisa, Pisa, Italy; 3 Department of Political Science, University of Bari, Bari, Italy; 4 Department of Economics and Law, University of Rome “La Sapienza”, Rome, Italy; University of Salerno, ITALY

## Abstract

Digital labour platforms are reshaping the world of work across a wide range of sectors, offering greater flexibility and accessibility than traditional labour markets. However, existing research suggests that platform work is often associated with low-quality working conditions and may exacerbate inequalities. This study examines the economic and social dimensions of digital platform labour in Italy—a country characterised by labour market fragmentation and the widespread use of non-standard employment—using official survey data collected in 2018 and 2021. Applying advanced machine learning (ML) and explainable artificial intelligence (XAI) techniques, the analysis explores the demographic, occupational, and economic factors that predict participation in platform work and drive segmentation within the platform workforce. The findings reveal that platform work in Italy is a heterogeneous and stratified phenomenon, deeply embedded in longstanding labour market fragmentation and regional disparities. Economic vulnerability is concentrated not among the youngest workers, as often suggested in the literature, but among older or more established individuals facing job instability, underemployment, or declining income from traditional occupations. Moreover, the analysis reveals that platform work is associated with structural vulnerabilities typical of non-standard employment, including unstable contracts, gender inequalities, and economic insecurity, and it primarily functions as a compensatory mechanism to supplement insufficient earnings from precarious jobs. Among jobseekers, engagement with platforms is more likely among younger individuals experiencing moderate—rather than severe—financial strain, suggesting that platform work is not generally perceived as a last-resort strategy but rather as a temporary or adaptive response to limited labour market opportunities. The COVID-19 pandemic further intensified these dynamics, acting as a catalyst for workers experiencing economic and social stress. During this period, platform work expanded as a fallback option for the unemployed, providing an informal buffer amid declining employment opportunities and persistent income insecurity.

## 1. Introduction

Digitalisation, epitomised by the diffusion of digital platforms and, more recently, generative Artificial Intelligence (AI), has radically transformed the way we communicate, produce, consume and work. Labour markets have been transformed in several ways: routine jobs have been replaced, existing occupations have been digitised, new jobs have been created, and algorithmic systems have been adopted for managing industrial relations [1]. This transformation has fuelled a growing debate, reflected in a mixed empirical literature [2–5]), oscillating between optimism about flexibility, innovation, and new employment opportunities, as supported by positive yet quantitatively negligible effects of AI on employment and wages of high-tech areas and sectors rewarding high-skilled occupations; and concerns over job loss, raising the risk of polarisation and inequalities, and the spread of precarious, opaque forms of digital labour.

Digital platforms constitute a paradigmatic case of this ambivalent transformation in the world of work. They operate as online intermediaries that connect providers of services or goods (commonly referred to as “platform workers” in case of digital labour platforms) with clients or businesses that purchase them [6–8]. On the other hand, part of the rise in non-standard work—which wholly or partially lacks safeguards against the socio-economic risks that have become widespread in recent years—can be attributed to the emergence of platforms. Despite their heterogeneity, digital platforms can be understood as a vast and dynamic ecosystem encompassing a wide array of activities, business models, and sectors that span global, regional, and local scales. Although a variety of terms have been used to describe platform workers—such as “gig workers”, “online workers”, and “digital platform workers”—their activities can be broadly divided into two main categories, depending on the nature of the work performed and the type of digital platform involved [9–11]. *Web-based platforms* mediate and organise labour services performed remotely, including both online freelancing and crowdwork, with tasks ranging from high-skill software development, data analysis, and graphic design to routine microtasks. *Location-based platforms*, by contrast, enable the provision of services in a specific physical location. These typically include transportation, care, retail, and delivery activities, which are coordinated and assigned through digital or mobile applications but carried out in the physical world. In both settings, platform workers are individuals who offer their labour in exchange for remuneration. However, there is still no clear consensus regarding their contractual status (e.g., whether they should be classified as employees or as self-employed). This legal ambiguity has significant implications for their access to labour rights and social protection [8].

Focusing on plaform workers from both theoretical and empirical perspectives, the literature has, on the one hand, examined how the expansion of the platform economy has facilitated labour market entry for vulnerable groups, including immigrants [12], individuals with disabilities [13], and workers in the Global South [14]. The rapid growth of platforms reflects their capacity to open new markets and generate novel forms of work and income, particularly for those with limited alternatives and restricted access to social protection [9]. This is driven by lower entry barriers [15,16], as well as by the demand for flexibility in working arrangements and employment conditions among certain vulnerable groups, reflecting the specific characteristics of their personal circumstances [17]. On the other hand, the literature has also emphasised the precarious working conditions of platform workers, which contribute to greater economic insecurity [18], particularly through low pay and the volatility of working hours and earnings [19–21]. Research in this field highlights how the organisation and performance of work through digital platforms pose significant challenges to ensuring decent wages. Platform workers often experience precarious employment relationships and face considerable uncertainty regarding income stability and protection against social risks [22]. These conditions are further exacerbated by a distinctive feature of digital platforms, namely algorithmic management. Evidence shows that the systems governing workers’ activities can intensify their vulnerability: platform workers are frequently isolated, subject to continuous monitoring, and have limited capacity to understand or challenge decisions made by the digital systems that manage their work [8,23]. Several contributions within this stream of literature also point out that, by encoding potentially discriminatory rules and learning from biased data, digital platforms risk reproducing and amplifying existing social and economic inequalities. Moreover, the extensive collection, storage, and processing of workers’ data reconfigure control and power relations in the workplace, embedding new forms of algorithmic governance within the digital labour process [24–27].

A further strand of research has examined the impact of digital platforms on the structure of the labour market. The phenomenon of segmentation has long been recognised by non-mainstream economists and sociologists [28,29] as a structural process characterising contemporary economies, particularly in Mediterranean countries occupying subordinate positions within global value chains and exhibiting a persistent demand for flexible and low-skilled labour [30]. In this context, recent digitalisation has further intensified labour market polarisation [31]. The expansion of the platform economy offers new opportunities to analyse stratification in labour markets. However, with the exception of a limited number of case studies [32], stratification within platform work itself remains largely underexplored, as does its interaction with other forms of non-standard employment and unemployment. This gap in the literature reflects the broader difficulty of capturing the fragmented and elusive nature of platform work.

The Italian case, which is the focus of this study, is a particularly suitable context for investigating these issues. In Italy, non-standard work has expanded more rapidly than in most European countries, accompanied by stagnant wages, increased reliance on temporary and involuntary part-time work, and deteriorating conditions for vulnerable groups [33]. At the same time, in Italy digital platforms have to a significant extent penetrated sectors already characterised by precariousness, such as retail, tourism, and hospitality [34]. In these sectors, platform workers exhibit significantly higher levels of economic vulnerability compared to other employment categories [18], whilst the technological dependence of businesses on platforms in these sectors (e.g., hotels rely heavily on booking services, which are often operated as monopolies) can result in economic pressure (e.g., rising fees for access to digital services) that may further exacerbate job insecurity [35].

### 1.1. Scope and structure of the study

Against this backdrop, and notwithstanding the growing body of research on the subject, several key questions remain unresolved. Empirical evidence on the social and economic determinants of participation in platform work, as well as its implications for vulnerability, remains incomplete and inconclusive. This is also due to the so-called *“digital paradox”*: while platforms allow unprecedented transparency by tracing and predicting productive and economic behaviours, their workers are trapped in an opaque digital black box, bearing most socio-economic risks and finding it difficult to understand and challenge managerial (algorithmic) decisions [36]. This study presents new, granular empirical evidence on the demographic, social, and economic characteristics of individuals engaged in platform work, thereby contributing to the existing literature on whether platform work represents a new and distinct form of precarious labour in Italy, one that deepens the vulnerabilities already embedded in fragmented labour markets. In doing so, it offers an insightful comparison with the rest of the workforce, examines whether and to what extent significant differences exist among platform workers themselves (i.e., whether they constitute a homogeneous population or comprise distinct segments differentiated by socio-economic and occupational characteristics), and analyses the consequences of the pandemic shock. From this perspective, we explicitly engage with sociological contributions that examine how, and to what extent, digital platforms contribute to labour market dualism and segmentation by reinforcing the presence of a disadvantaged segment of the labour market [32]. We pay particular attention to the intersection between platform work and other forms of non-standard employment and unemployment.

To pursue this goal, the study adopts a complex systems perspective and draws on unique survey data to analyse the socio-economic dimensions of platform work in Italy, focusing on key individual, occupational, familial, and geographical characteristics. By exploiting the capacity of Machine Learning (ML) methods to capture the contribution of granular elements and their interactions—typically overlooked in aggregate analyses or conventional econometric approaches—this study provides a more detailed, feature-specific, and informative characterisation of platform work than is currently available [18]. More specifically, the mapping of platform workers relies on a set of demographic and socio-economic variables. Age, gender, educational attainment, household composition, country of birth, and place of residence (in terms of degree of urbanization) have been shown to be significant predictors of participation in digital platform work [37]. Previous empirical research also highlights the role of labour market status, suggesting that employees in standard forms of employment—namely those with open-ended contracts—are less likely to engage in platform work [37]. For this reason, we explicitly include employment status in our analysis, such as occupation, contractual arrangement, sector of activity, and part-time employment. Moreover, existing literature suggests that participation in digital platform work is socially stratified, with more vulnerable workers disproportionately represented among platform workers. Individuals with limited access to standard employment—due to labour market exclusion, precarious employment histories, or lack of social protection—are more likely to enter platform work, which is characterized by low entry barriers but also by instability and weak labour protections [38–41]. This results in a concentration of economically vulnerable workers within digital platforms. Therefore, we include in the analysis a set of variables proxying economic vulnerabilities, as described in table A1 in S1 Appendix.

**Fig 1:**
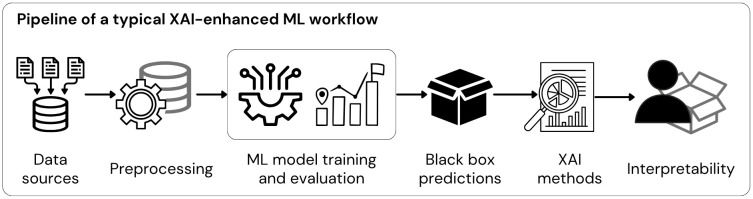
Simplified representation of the methodological pipeline. Starting from multiple data sources, the workflow proceeds through data preprocessing (including feature selection, imputation of missing values, dimensionality reduction of high-cardinality variables and analysis of correlated variables), model training and evaluation, and black-box prediction generation, followed by the application of an XAI methods to produce interpretable outputs.

Methodologically, we employ ML techniques to identify complex, non-linear relationships in the data, complementing traditional hypothesis-driven approaches with a data-driven perspective that reduces a priori modelling assumptions. This is particularly relevant given the high granularity of the dataset and the strong heterogeneity of platform workers, which call for flexible tools capable of detecting subtle patterns across multiple dimensions. To ensure interpretability, we integrate explainable AI (XAI) methods [42], which provide transparency into model predictions and strengthen the reliability of our findings. The complete pipeline of the employed methodology is depicted graphically in Fig 1.

The analysis is based on three main research questions (RQs):

- **RQ1 Mapping platform workers in Italy**: What are the demographic and socio-economic characteristics of platform workers in Italy and to what degree are they exposed to economic vulnerability?
- **RQ2 Platform work and non-standard employment**: How does platform work intersect with other forms of non-standard employment and do platform workers represent an even more vulnerable subgroup within this category?
- **RQ3 Platform work and unemployment**: How does platform work intersect with unemployment trajectories, and which factors predict jobseekers’ participation in platform work as an alternative to traditional employment?

By answering RQ1, we aim at advancing knowledge concerning the distinctive features of platform work (i.e., *who are the platform workers in Italy? Why are some individuals and social groups more likely to join platform work than others?*) and their relationship with economic vulnerability. Although this is a widely debated topic [9,43,44], the available empirical evidence is still relatively scarce and based on qualitative analysis. RQ2 allows linking the literature on platform work with the larger debate on non-standard work, precarisation and its broad socio-economic implications. In particular, we test whether the diffusion of digital platforms is accelerating the process of precarisation observed in many advanced economies since the late 1990s [18,45]; or, in turn, if a new peculiar form of precarious workforce is emerging on top of the existing one. Finally, RQ3 links the analysis of platform workers with one of the main targets of labour policy: the unemployed. In so doing, we assess whether the combination of organisational fragmentation (i.e., platform workers being scattered and often isolated, most of the time interacting exclusively with the algorithm that assigns and monitors their tasks), digital monitoring (i.e., surveillance-based algorithmic management tools) and institutional weaknesses (i.e., non-standard contracts and poor protections against socio-economic risks) renders platform workers even more vulnerable than the unemployed, creating new challenges for social and labour policies. Drawing on data from both 2018 and 2021, we also examine how these patterns evolved in the wake of the COVID-19 pandemic, under the hypothesis that its structural effects on social life, economic organisation, market dynamics, and especially the expansion of digital markets have had a significant impact on platform work.

The remainder of this paper is structured as follows: Section 2 describes the data source and the ML and XAI methodologies applied. Section 3 presents and discusses findings organised around the three research questions. Specifically, Section 3.1 maps the demographic and socio-economic profile of digital platform workers (RQ1), Section 3.2 explores the connections between platform work and non-standard employment (RQ2), and Section 3.3 analyses how platform work intersects with unemployment trajectories (RQ3). Section 3.4 further describe the role of the pandemic crisis in reshaping platform labour. Section 4 reviews the findings across all research questions, while 5 closes the paper presenting its limitations, policy implications, and directions for future research.

## 2. Materials and methods

### 2.1. Data

This study utilises third-party, fully anonymised data from the *“Participation, Labour, Unemployment, Survey”* (PLUS), which is a recurring labour-market survey designed and administered by the Italian National Institute for Public Policy Analysis (INAPP). The analyses in this paper draw on the 2018 and 2021 waves of the INAPP PLUS survey, currently the latest waves with accessible and reliable microdata on platform workers. Please note that, although a 2022 wave exists, INAPP did not release any data related to platform work for that year due to severe data quality issues, and no subsequent waves has been published to date [46,47]. The surveys, included in the Italian National Statistical Programme under code IAP-00004, were conducted by INAPP independently of this research, following strict ethical, methodological, and legal standards applicable to official statistics, including full compliance with the EU General Data Protection Regulation (GDPR). Participants were informed about the survey’s purpose and data-processing practices through an official information letter issued by INAPP, in accordance with Article 13 of GDPR, and the data collection was successively performed through the CATI (Computer-Assisted Telephone Interviewing) methodology. All personal identifiers were removed before data were made available to researchers, and no re-identification is possible. Although not publicly available due to confidentiality restrictions, the datasets may be requested from INAPP for research purposes and are provided free of charge in fully anonymised form in accordance with national statistical and privacy standards. The authors did not participate in data collection and acted solely as secondary data users. Refer to https://www.inapp.gov.it/en/surveys/periodic-surveys/participation-labour-unemployment-survey-plus for a comprehensive description of the data source and https://www.inapp.gov.it/en/surveys/microdata for detailed information on how to gain access to the data.

The primary objective of the PLUS survey is to deliver reliable estimates of labour market characteristics that are only partially addressed by other surveys. Indeed, it collects detailed information not just on employment status and history but also on income, family background, education, household characteristics, health, and transitions over time, including effects of new technologies and shocks such as the pandemic. Importantly, it also includes an *ad hoc* module on platform work, an element that is lacking in other large-scale surveys, which enables comparisons between these workers and the broader workforce. This facilitate the analysis of the socio-economic landscape of digital platform work in a nationally representative setting. Specifically, the PLUS survey defines a digital labour platform as a *“a website or mobile app that connects directly with people who request their services. These platforms (e.g., Deliveroo or Glovo) require workers to create a user profile to find and accept tasks or assignments and to receive payment once the service has been completed”* and a platform worker as anyone who answers affirmatively to the following question: *“Currently, or in 2020, have you earned money by accepting jobs through this type of website or mobile app—for example, delivering meals or products to someone’s home, cleaning someone’s house, or performing tasks or work assignments obtained online?”*.

The survey design is based on a stratified sampling model of the Italian population, with strata defined by region, type of municipality, age, gender, and employment status. The surveys cover representative samples of 45,000 individuals in 2018 and 46,282 individuals in 2021, all aged 18–74. Within these samples, 222 and 492 respondents, respectively, reported engaging in digital platform work. After applying survey weights, these figures correspond to an estimated 785,480 platform workers out of a total reference population of 86,687,651 individuals across the two years. Note that our definition of platform workers disregarded the formal requirement of a written contract, since in these contexts there is often no legal employment framework in place. The survey further distinguishes between the following activities: delivering products or packages (16%), delivering meals (27%), performing online tasks (e.g., data entry, software development; 29% overall), domestic work (8%), driving someone by car (10%) and other unspecified activities (9%). However, due to the limited sample size of platform workers, we could not analytically differentiate between these activity types. Consequently, our analysis aggregates all forms of platform work (i.e., both location- and web-based) into a single category. This approach allows us to identify common structural features shared across different types of task, including precarious conditions, economic vulnerability, and limited protections. As a result, our findings offer a conservative estimate of the vulnerabilities that characterise platform workers as a whole (working conditions even worse than those documented could emerge if the focus were limited to the most vulnerable groups, such as food delivery riders). Moreover, while the survey design does not allow us to accurately distinguish between primary versus secondary platform income, we can provide descriptive statistics on platform workers’ labour market status: applying sample weights, 63% of platform workers report having traditional employment, 15% are job seekers, and 22% are inactive.

In contrast to more general datasets on labour, PLUS can be deemed “intersectional” since it provides data disaggregated by several social categories (e.g., gender, age, place of origin, and medical condition). This breakdown is of particular interest since it exposes multiple intertwined drivers of labour precarisation [48–50]. In this context, an intersectional approach to data allows identifying inequalities both within and between groups of individuals, contingent upon the interplay of several aspects of a person’s identity.

#### 2.1.1. Variable preprocessing.

Data processing is organised as follows. We started with a hypothesis‐driven variable selection focusing on demographic characteristics, educational attainment, employment contract type, household composition, working conditions, and regional indicators. Next, we cleaned the selected variables by first introducing the new category ’Not applicable’ to account for survey items that were not relevant to certain respondents (e.g., questions concerning contractual characteristics posed to individuals who were unemployed); for the few remaining missing entries, we applied a *k*-Nearest Neighbours imputation algorithm to preserve underlying distributional properties. Third, to mitigate overfitting and improve model interpretability, we performed dimensionality reduction on high-cardinality categorical features, particularly on those with highly skewed level frequencies, by collapsing rare levels into a single category or merging semantically similar groups. Finally, we performed data-driven variable selection based on the exploration of Pearson correlation, Variance Inflation Factor, and Chi-square tests to retain only those predictors demonstrating significant associations and minimal multicollinearity.

Note that each subsequent ML task is trained on a customised subset of these processed variables. A summary of these subsamples, including global information on dimensionality and feature composition, as well as the specific ML pipeline utilised for each research question, is presented in Table 1. The full list of variables and corresponding values used in this work is reported in A1 in S1 Appendix.

**Table 1:** Description of the data and methodology employed to investigate each research question.

Research question	Subsample	No. samples	No. fueatures	Class imbalance	Variables	ML algorithm	Interpretation method
**RQ1. DPW mapping**	Employed DPW	368	35	–	Demographic, socio-economic and **platform work**	*k*-means clustering	Feature-value distribution, Decision trees
	Unemployed DPW	343	28	–			
**RQ2. DPW & Non-standard employment**	Non-standard workers & DPW	15984 *2021: 8779* *2018: 7205*	31	4.5%	Demographic, socio-economic and **employment**	CatBoost	SHAP
RQ3. DPW & Unemployment^*^	Job-seekers below 50 years old	10833 *2018: 6672* *2021: 4161*	22	1.5%	Demographic, socio-economic and **unemployment**	XGBoost	SHAP

DPW = Digital Platform Workers. Class imbalance refers to the proportion of DPW in the subsample under analysis.

* In this row, dataset size refers to the pre-processed sample; after applying a combination of undersampling and oversampling, the analysis of RQ3 was conducted on a dataset of 11,044×22 observations (2021: 6,831; 2018: 4,213) with a class imbalance of 3.8%.

### 2.2. Machine learning methods

All analyses were conducted using Python, employing standard machine learning and explainable AI libraries. The former were selected based on the distinct scopes of the research questions: specifically, unsupervised methods (i.e., cluster analysis) for RQ1 and supervised learning methods (i.e., binary classification) for RQ2 and RQ3. The complete code used for data processing, modelling, and visualisation is openly available at https://github.com/cpunzi/Digital-Platform-Work-Italy.

#### 2.2.1. Cluster analysis.

In order to conduct a comprehensive cluster analysis of digital platform workers in Italy, we compared different clustering methods and determined the most effective one using the silhouette coefficient, the Davies-Bouldin score and the Calinski and Harabasz score. The algorithms taken into consideration include *k*-means with different numbers of clusters, hierarchical clustering with different linkage methods, including complete, average, single, and ward, HDBSCAN and spectral clustering.

#### 2.2.2. Classification models.

We developed two binary classification models: the first aimed at examining the relationship between platform work and non-standard employment, and the second focused on identifying the factors influencing job seekers under the age of 50 to participate in platform work. These two models are trained on different subsamples of the PLUS dataset with respect to both the individuals and variables included. Nevertheless, their development (training, testing and interpretation) is analogous. In both cases, the dependent variable captures participation in platform work.

We utilised a variety of machine learning algorithms, including Decision Tree, Random Forest, CatBoost, XGBoost, MLP and TabNet, to build our models. The datasets were split into training, validation, and test sets using stratified random sampling to preserve class distributions. First, the data was divided into training (80%) and test (20%) sets; subsequently, the training set has been further split into training (64%) and validation (16%) subsets. All splits were performed with a fixed random seed to ensure reproducibility, and stratification was applied based on the target variable in both splitting steps, namely, being a platform worker. Sample weights were carried through consistently across all partitions. Hyperparameter tuning was performed using GridSearchCV in combination to optimise the performance of each classifier, which we measured through the F_1_-score given the high imbalance of the classification task. In this phase, models were trained and evaluated using stratified *k*-fold cross-validation to ensure robustness and mitigate overfitting. The final model was selected based on its cross-validation performance, and its predictive power was further validated on the test set (see B2 in S1 Appendix). For RQ2, the classifier with the greatest F_1_-score was CatBoost, while the optimal classification model for RQ3 proved to be XGBoost. Importantly, we also estimated baseline logistic regression (Logit) models for both research questions. As reported in B3 and B4 in S1 Appendix, these models exhibit consistently lower performance across all evaluation metrics, confirming the presence of non-linearities and complex interactions that are not fully captured by linear specifications. Such comparison confirms that our ML strategy more effectively identifies trends and relationships in the data than traditional approaches, such as the logit model. Full details on the training and evaluation pipeline, including a statistical assessment using crucial difference diagrams, are included in Section B in S1 Appendix.

### 2.3. Explainable AI models

To enhance the interpretability of the ML models used in this study, different techniques were employed to break down the outputs of clustering and classification tasks, thereby enhancing algorithmic transparency and facilitating an understanding of the socio-economic patterns underlying the examined research questions.

In order to characterise the subgroups of digital platform workers (RQ1), we analysed the distribution of feature values across groups. Cluster membership was also predicted through binary decision tree classifiers, which are inherently explainable due to their transparent structure of hierarchical decision rules. Furthermore, feature importance scores for this prediction task were calculated to accurately delineate the distribution of relevant feature values across clusters.

The interpretability framework for the two classification tasks addressing RQ2 and RQ3 relies on the analysis of global SHAP (SHapley Additive exPlanations) values. SHAP [51] is a model-agnostic game-theoretic explainability method that attributes the output of a model to its input features by computing their marginal contributions, thus quantifying the contribution of individual features to model predictions. To ground the analysis of SHAP values on robust statistical evaluation, we further verify the feature relevance scores through StableSHAP [52], a novel technique that enhances SHAP via a sampling procedure that finds the top-*K* most important features, possibly with their relative ordering, with high probability guarantees. For a comprehensive description of the utilised explainability technique, please refer to Section C.2 in S1 Appendix. Notably, while SHAP (SHapley Additive exPlanations) is a powerful tool for interpreting predictive models by making feature correlations transparent, it does not provide causal inferences [53]. Feature attribution scores are computed under the assumption that the underlying data distribution remains fixed, meaning they cannot predict how manipulating a feature would change an outcome, nor do they account for confounding variables. Therefore, SHAP alone is unsuitable for establishing causal relationships and its results must be interpreted strictly as correlational explanations of the model’s predictions.

## 3. Results

### 3.1. Demographic and socio-economic mapping of digital platform workers in Italy

**Fig 2:**
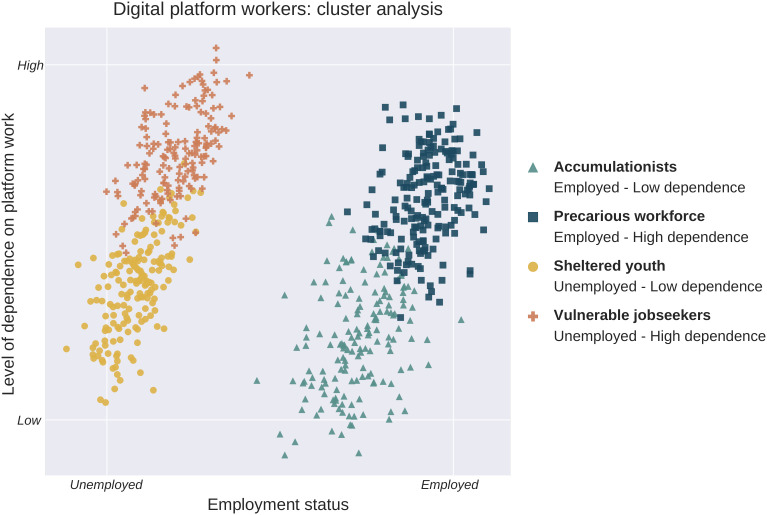
Cluster of platform workers. Visualisation of the four distinct profiles of digital platform workers in Italy, identified through cluster analysis, positioned along two main dimensions: employment status (employed vs. unemployed) and level of dependence on platform work (low vs. high).

Who are digital platform workers in Italy? Which socio-economic factors shape their condition? Our clustering analysis reveals that this component of the Italian workforce is far from homogeneous. Rather than constituting a cohesive occupational group, platform workers occupy diverse and fragmented positions within the Italian labour market, reflecting intersecting inequalities of employment status and socio-economic vulnerability. Two main structural dimensions underpin this heterogeneity: (i) workers’ *relationship to traditional employment*, namely, whether they are employed or not; and (ii) their *degree of dependence on platform earnings*, ranging from essential income sources to merely occasional or complementary roles. These two axes intersect with broader dynamics of precarity, economic necessity, and personal and family circumstances, while also being strongly shaped by intersectional attributes such as gender, age, and geographical location. Crossing the two main dimensions, we identified four clusters of comparable dimensions, each corresponding to a distinct profile of digital platform worker (refer to Fig 2 for a graphical representation). Table 2 presents a synthetic profile of platform workers across clusters, reporting only the most representative characteristics and variables that meaningfully differentiate groups. The full set of descriptive statistics, including modes, means and standard deviations for all variables by cluster, is provided in C5 in S1 Appendix.

**Table 2:** Representative characteristics of platform workers by cluster. The table reports the most typical values within each cluster to provide a synthetic profile of workers’ socio-demographic and economic conditions. Only variables displaying meaningful variation across clusters are included.

	Sheltered youth	Accumulationists	Vulnerable jobseekers	Precarious workforce
Geographical area	Center and South	North	North	South and Islands
Age group	25-29	25-29	30-39	40-49
Marital status	Single	Single	Couple	Couple
Family type	Child of a couple	Child of a couple	Couple with children	Couple with children
Children	No	No	Yes	Yes
Caregiving	No	No	Yes (regularly)	Yes (regularly)
Years since last qualification	3-5 years	10-20 years	20 + years	20 + years
Occupational status	Inactive	Employed	Jobseeker	Employed
Part-time status	Not employed	Incompatible contract	Not employed	Yes (voluntary)
Side job	Not employed	No	Not employed	Yes
Monthly net income	Not employed	Less than €1,000	Not employed	Less than €1,000
Skill mismatch	Not employed	About the same	Not employed	Slightly higher
Years since first job	Not employed	5-10 years	Not employed	20 + years
Occupation (also previous)	Highly specialised	Not employed	Office work	Office work
Household income	€1,501–2,000	€2,001–3,000	€1,001–1,500	€1,501–2,000
Household food expenditure	25–50%	25–50%	25–50%	10-25%
Household mortgage expenditure	0–10%	0–10%	0–10%	10-25%
Maximum extraordinary expense	€300–800	€300–800	Less than €300	€300–800
Postponement of medical treatment	No	No	Yes	Yes
Relevance of platform work	Convenient but not necessary	Convenient but not necessary	Important but not essential	Essential to meet basic needs
Online sales	No	No	Yes	Yes
Online home-sharing	No	No	No	Yes

The two clusters characterised by high economic dependence on platform earnings reveal the persistence of structural fragility across both employment and unemployment contexts, where platform work increasingly operates as an informal safety net for economically vulnerable households. These groups are composed predominantly of older men (well above the average age typically reported in the literature) who often shoulder caregiving responsibilities for children and for relatives or acquaintances with permanent reductions in autonomy. Both clusters exhibit clear signs of economic strain, including the postponement of medical treatments and limited capacity to absorb unforeseen expenses. Their reliance on the digital platforms is often reinforced by complementary income sources such as online sales and home-sharing. The COVID-19 pandemic further deepened their vulnerability, causing job losses and lasting financial hardship for many. The two clusters differ mainly in their employment status and geography: the **precarious workforce** includes individuals concentrated in Southern Italy and the islands, typically engaged in part-time jobs with very few hours and low pay despite long work histories; conversely, the **vulnerable jobseeker** is primarily located in Northern Italy, comprising individuals, including non-Italian workers, with prior employment experience who are currently job-seeking. Together, they capture two facets of the same phenomenon: workers pushed toward platform labour not by choice but by necessity.

In contrast, the two clusters with low dependence on platform work show that digital labour also attracts individuals with comparatively stable socio-economic conditions. These groups are predominantly younger (under 30 years old, consistent with the age ranges typically emphasised in the literature) and are often still living with their families of origin, without any caregiving responsibilities. For them, platform work represents a marginal or exploratory activity rather than a coping mechanism against economic hardship. Again, employment status and geographical differences further distinguish the two clusters: the **accumulationists** are concentrated in Northern Italy, holding stable full-time jobs that make platform work largely supplementary; in contrast, the **sheltered youth** is primarily situated in Southern Italy and are inactive rather than actively seeking employment, unlike the cluster of unemployed with high dependence on platform work.

Crucially, these findings challenge a dominant assumption in the literature that younger workers are the most represented and vulnerable within the platform economy. In the Italian context, marked by a peculiar, fragmented and structurally precarious labour market, economic fragility instead tends to cluster among older or more established workers who experience job instability, underemployment, or declining income from traditional forms of work.

Taken together, the four clusters suggest that digital platform work in Italy does not represent a uniformly precarious form of employment but rather a heterogeneous phenomenon shaped by pre-existing inequalities in the labour market. Only the two high-dependence clusters, comprising older workers with caregiving responsibilities, show clear signs of socio-economic fragility together with contrasting geographical patterns (employed in the South, unemployed in the North). Conversely, the low-dependence clusters, mainly composed of younger individuals living with their families and exhibiting relative economic security, engage with platforms in a marginal or exploratory way. These findings indicate that platform work may amplify existing structural divides rather than creating an entirely new form of precariousness, embedding digital labour within the broader fragmentation and regional asymmetries of the Italian labour market.

### 3.2. Platform work and non-standard employment

**Fig 3:**
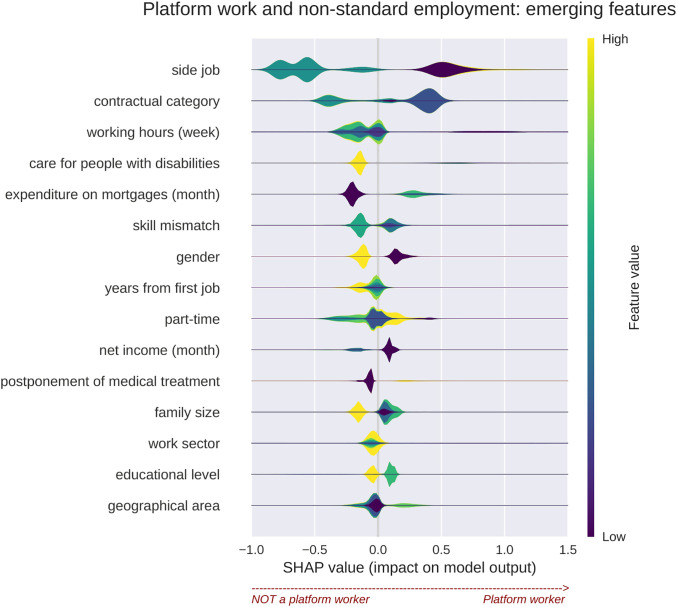
Top factors predicting engagement with the digital platform workforce among non-standard workers (RQ2). Each row shows the distribution of individual SHAP values, coloured according to the colour bar on the right from high (yellow) to low (purple) feature values, where the mean absolute SHAP value represents the average magnitude of that feature’s impact on the model’s prediction across all samples. Positive SHAP values indicate a push toward classifying an individual as a platform worker. For categorical variables, the specific category-to-colour mapping is shown directly beneath each variable name along the left *y*-axis.

How does platform work intersect with other forms of non-standard employment? Do platform workers constitute a particularly vulnerable subgroup? Rather than standing apart from other non-standard forms of employment, platform work emerges as an intensified expression of the same structural fragilities linked to unstable contractual arrangements, economic vulnerability, and gendered inequalities. The feature importance rankings enabled the interpretation of the characteristics that the ML model most strongly associates with platform worker status within the wider category of non-standard labour. The global SHAP summary plot is displayed in Fig 3, while a comparison with other feature ranking methods and a visualisation about single feature effects on model prediction are both reported in Section C.2 in S1 Appendix.

Two dimensions stand out most clearly: employment contracts and income diversification. Platform workers are typically associated with multiple and unstable income sources, often holding a secondary job in addition to part-time or fixed-term employment. This pattern is consistent with the interpretation that platform work may serve as a compensatory mechanism, supplementing insufficient earnings from traditional employment rather than providing an autonomous livelihood, though the model itself does not test this causal relation. Conversely, self-employed workers, particularly holders of VAT numbers, show a negative correlation with platform work in the model’s predictions. VAT holders also rank as the least precarious within the non-standard workforce according to the features examined: while they may still experience income instability and limited social protection, they typically retain greater autonomy in organising their work and managing their earnings without the need for additional occupations. Weekly working hours display a non-linear pattern: unemployment or minimal work (less than 16 hours per week) shows a positive correlation with being a platform worker, whereas longer hours typically exhibit a negative correlation, except in particular instances of part-time permanent employment (if up to 20 hours) or fixed-term employment (if above 30 hours), where a weak positive correlation remains.

Beyond contractual and working status, a range of socio-economic vulnerability markers also show associations with platform workers status. These include high mortgage expenditure, caring for relatives or acquaintances with a permanent reduction in autonomy, low income and the need to defer medical treatments (including dental care) in the last year due to financial constraints, pointing to the weight of economic pressures in shaping labour trajectories. Skill mismatch and gender also play a role: men and overqualified workers are slightly more likely to be classified as platform workers by the model, though these effects are less pronounced. Sectoral affiliation, by contrast, shows limited influence, with only slight positive effects observed in wholesale and retail commerce, as well as in professional, scientific, and technical services, suggesting that vulnerability-related factors cut across occupational domains.

Taken together, these findings reinforce the depiction of platform workers as the most vulnerable segment of the non-standard workforce, characterised by unstable contracts, multiple job dependencies, and financial strain.

### 3.3. Platform work and unemployment: contemporary pathways beyond the traditional job market

**Fig 4:**
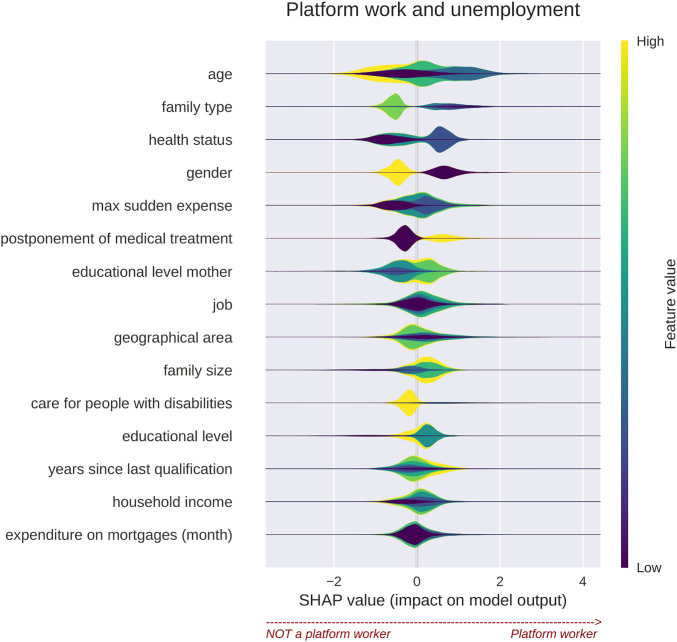
Top factors that drive job-seekers under 50 to pursue employment opportunities on digital platforms (RQ3). Each row shows the distribution of individual SHAP values, coloured according to the colour bar on the right from high (yellow) to low (purple) feature values, where the mean absolute SHAP value represents the average magnitude of that feature’s impact on the model’s prediction across all samples. Positive SHAP values indicate a push toward classifying an individual as a platform worker. For categorical variables, the specific category-to-colour mapping is shown directly beneath each variable name along the left *y*-axis.

The analysis of jobseekers under 50 provides important insights into how digital platform work intersects with unemployment trajectories in Italy. Using binary classification and SHAP-based model interpretation, we identified the variables that, according to the ML model, most distinguish jobseekers who turn to platform work from those who do not, thereby exposing the multidimensional factors associated with this form of labour participation. Although establishing the precise order of feature relevance proved challenging (see Section C in S1 Appendix), a number of recurring predictors could still be identified. For a visual representation, refer to the global SHAP summary plot displayed in Fig 4 and the scatter plots representing single feature effects on model prediction reported in Section C in S1 Appendix.

Platform work is correlated with well-defined socio-economic features and is more asymmetrically distributed across regions as compared to the wider pool of job seekers. Men, individuals aged 25–30, and residents of central and northern Italy show the highest positive correlations with platform work, while women, older jobseekers, and those located in the South are less represented. Family configuration also plays a central role: single individuals and couples with children are more frequently classified as platform workers than young adults still supported by their families.

Signs of socio-economic vulnerability also emerge clearly. Jobseekers who had to postpone medical treatments (including dental care) for financial reasons, or who report a limited ability to cope with sudden expenses, exhibit a positive association with platform work. Moreover, caring for relatives or acquaintances with a permanent reduction in autonomy also acts as a strong positive predictor in the ML model. However, our analysis also indicates that platform work is not positively correlated with situations of severe economic distress. This is shown, for instance, by the fact that both very low family income and high shares of household income devoted to mortgage repayments act as negative predictors. Although the model does not test causal links directly, this pattern is consistent with the interpretation that platform work is more often adopted as a buffer for those under moderate, rather than extreme, economic pressure. This intermediate positioning may also reflect the coexistence of individuals with varying levels of dependence on platform earnings within the unemployed population, an aspect that future research should investigate more explicitly.

Ultimately, occupational histories further illustrate the ambivalence of platform labour in unemployment trajectories. Individuals having both a high- and a low-skill background have an increased likelihood of being classified as engaging with platform work, pointing to a polarisation of entry points. For those who were never employed, outcomes vary by region: while in the North this status shows a positive association with platform participation, in the South this feature correlates negatively.

Overall, the findings of the analysis reveal that the features associated with platform workers aligns closely with the **vulnerable jobseeker** profile identified in the cluster analysis, yet with one important distinction: although the cluster comprises numerous adults and middle-aged individuals, the positive correlation with participation among jobseekers is markedly stronger for those who are younger, particularly within the 25–30 age bracket. This highlights a significant nuance: under comparable socio-economic conditions, and among jobseekers, younger individuals exhibit a stronger positive correlation with entering the platform workforce, despite this typically not being the case for this group in the broader population.

### 3.4. The role of the pandemic crisis: reshaping platform labour in the wake of Covid-19

Although data scarcity—particularly for the 2018 subsample—precluded the independent estimation of year-specific classification models, we included year as a binary predictive feature and conducted a post hoc cohort analysis comparing SHAP values across the two sampling years. As these years fall immediately before and after the COVID-19 shock, this comparison provides a lens through which to examine structural changes in platform work—both in scale and composition—potentially associated with shifts in demand (e.g., increased demand for digital services such as food delivery) and supply (e.g., workers turning to platforms as a relevant source of income) following the pandemic. In the Italian context, the pandemic represented not only a major disruption of medical and social life but also an unprecedented stress test for the economic system, with deep and lasting structural impacts on social functioning, economic organisation, and market dynamics, including an accelerated expansion of the digital platform economy [18,54].

**Fig 5:**
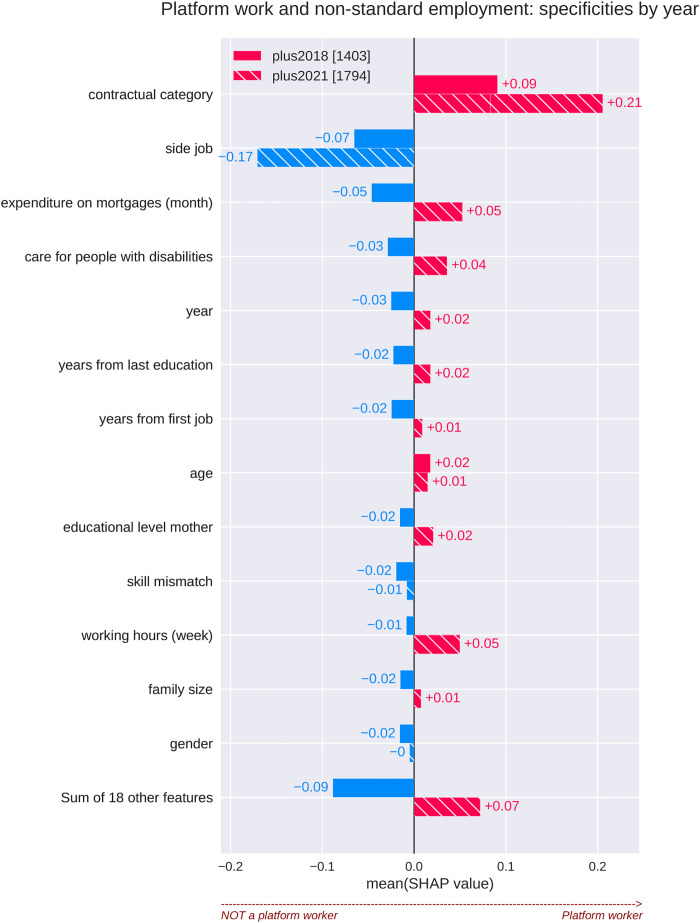
Mean SHAP cohort values showing how the year of observation (2018 *vs.* 2021) shifts the contribution of predictors to platform work with respect to RQ2. Features are ranked from top to bottom according to their mean absolute SHAP value. In each row, the upper bar corresponds to the average impact of that feature to the prediction of platform work for the sampling year 2018, while the lower dashed bar represents the same measure for the year 2021. Positive SHAP values (red) indicate a push toward classifying an individual as a platform worker.

When focusing on non-standard employment (RQ2, see Fig 5), the 2021 data reveal an intensification of the patterns observed in 2018, which is consistent with the interpretation found in the literature [54] that platform work increasingly functioned as a coping mechanism for workers under heightened economic and social strain. In 2021 more than 2018, many platform workers reported severe financial hardship, job loss, or lasting income damage that had not been fully recovered by the time of the survey. Reliance on income support measures, particularly emergency subsidies, was widespread. These findings confirm that the platform economy does not merely coexist with inequality but actively feeds on it, showing stronger correlations with those groups most exposed to precarity and marginalisation. In this sense, while we do not test any causal link in this work, our cohort comparative analysis support the idea that the pandemic acted as both a catalyst and a magnifier of the parasitic dynamics of digital capitalism, which thrives on existing social and labour market polarisation.

**Fig 6:**
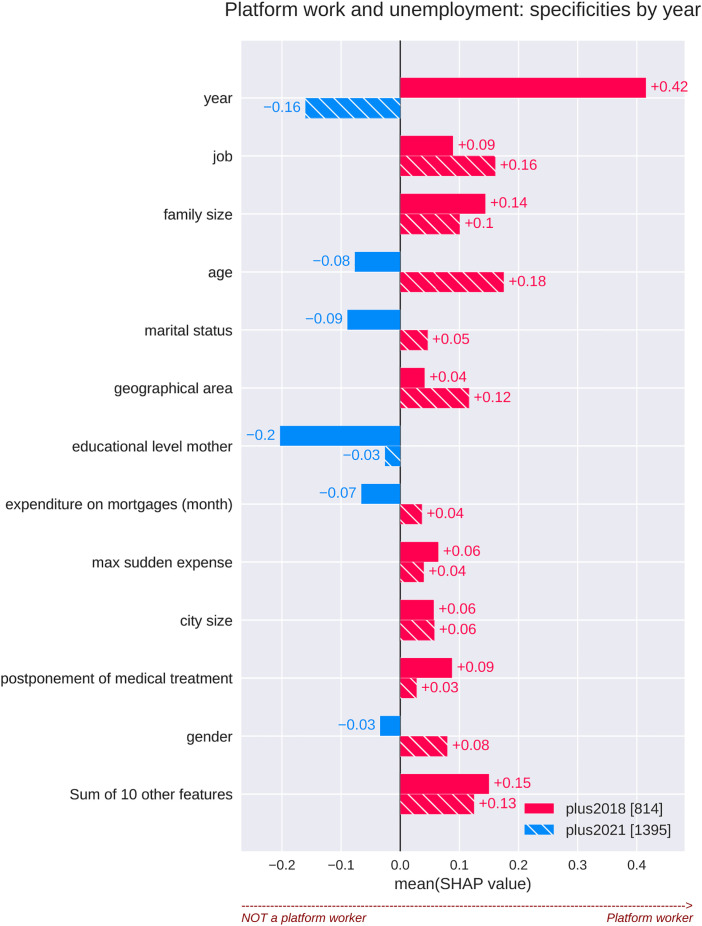
Mean SHAP cohort values showing how the year of observation (2018 *vs.* 2021) shifts the contribution of predictors to platform work with respect to RQ3. Features are ranked from top to bottom according to their mean absolute SHAP value. In each row, the upper bar corresponds to the average impact of that feature to the prediction of platform work for the sampling year 2018, while the lower dashed bar represents the same measure for the year 2021. Positive SHAP values (red) indicate a push toward classifying an individual as a platform worker.

A similar pattern emerges from the analysis of jobseekers (RQ3). Disaggregating the data by year (Fig 6) reveals that, in 2018, being a jobseeker was associated with a lower probability of being classified as a platform worker; by contrast, in 2021, the same condition became a strong positive predictor. This reversal indicates that, in the aftermath of COVID-19, platform work became more strongly correlated with a fallback option among unemployed individuals, potentially serving as an informal means of reintegration into economic activity within an environment of uncertainty and reduced labour market absorption. Alongside this structural shift, demographic factors, such as age, gender, marital status, and geographical area, gain weight in 2021, showing stronger associations of individual and territorial characteristics with the access to platforms during the crisis.

A refinement of the predictive model, which was obtained by training a classification model on the 2021 subsample only (an analogous model for 2018 could not be estimated due to insufficient data), reveals that an especially relevant characteristic of unemployed platform workers is previous work experience: the majority of unemployed platform workers in 2021 had held formal jobs before losing them due to layoffs, business closures, or dissatisfaction with working conditions. This pattern is consistent with the interpretation many entered the platform economy not as a first career choice but as a reactive adaptation to the shrinking opportunities and deteriorating job quality of traditional employment. This trend is corroborated by [43], who highlight that platform workers evaluate their platform work experiences in comparative terms with their previous roles in the traditional labour market. Moreover, these authors observe that economic precarity and dissatisfaction experienced in earlier employment have fostered a sense of acceptance toward the precarious, opaque, depersonalising, and alienating conditions imposed by platforms, justified by the ease of access and the potential earnings they offer.

In summary, the model’s patterns across 2018 and 2021 align with the hypothesis that the COVID-19 pandemic served as a structural catalyst for digital precariousness: while platform work offered short-term adaptability amid crisis, it simultaneously deepened long-standing inequalities and reinforced the dependence of vulnerable groups on unstable, digitally mediated forms of labour.

## 4. Discussion

By applying ML and XAI techniques to national survey data, this study has shed light on the complex interplay of demographic and socio-economic factors shaping platform work in Italy, highlighting the emergence of heterogeneous labour segments even within the platform workforce. In line with previous research, our analysis provides novel quantitative evidence on the precarious nature of platform work. We identify key socio-demographic and occupational characteristics shaping this segment of the labour market, pointing to a stratification of multiple layers of vulnerability. These vulnerabilities appear to have intensified following the COVID-19 pandemic, which, among other developments, coincided with an expansion of the digital economy.

More in detail, insights from our cluster analysis revealed that platform workers represent a heterogeneous group shaped by two main dimensions, namely, employment status and degree of dependence on platform earnings, intertwined with broader socio-economic vulnerabilities and demographic features. Crossing them, four distinct profiles emerge, ranging from the highly platform-dependent **precarious workforce** and **vulnerable jobseekers**, to the younger **accumulationists** and **sheltered youth** for whom platform labour remains marginal. This evidence challenges the widespread assumption that youth constitutes the most representative and vulnerable segment of the platform economy. Instead, in a labour market already marked by ageing, fragmentation and insecurity, vulnerability appears to concentrate among older or more experienced workers facing underemployment, declining job quality, or insufficient earnings from traditional work. Beyond the well-documented determinants of precarity (such as gender, financial strain, and employment instability), our analysis also identifies familial organisation as a distinctive factor. Workers that are highly dependent on platform income often carry significant caregiving responsibilities, including childcare and support for relatives or acquaintances with reduced autonomy. In line with [32], our results reveal that platform workers are highly heterogeneous: “high” and “low” segments often coexist, with the former typically using platform work to complement their income, and the latter experiencing greater vulnerabilities.

The analysis of the relationship between platform work and non-standard employment further shows that platform workers are associated with an intensified expression of the same structural fragilities (such as unstable contractual arrangements, economic vulnerability, and gendered inequalities) embedded in Italy’s labour market. A less pronounced trend differentiating platform workers exists among jobseekers, as they occupy an intermediate position on the vulnerability scale, which may also reflect the coexistence of individuals with varying degrees of dependence on platform earnings within the unemployed population, as previously observed through the cluster analysis.

Finally, we have shown that the patterns observed are consistent with the interpretation that the COVID-19 pandemic functioned as both a catalyst and an amplifier of these dynamics. For the employed, dependence on platform work was associated with a crucial coping mechanism in response to intensified financial pressure and weak public support systems, whereas for the unemployed, platforms increasingly functioned as an informal alternative, facilitating re-entry into economic activity amidst reduced labour absorption.

## 5. Conclusions

As a final remark, it is worth underscoring the unique value of the PLUS microdata. It remains the only nationally representative survey collecting, for a large sample size, rich multidimensional information and a dedicated module on the platform economy, enabling platform workers to be analysed in direct comparison with the wider labour force rather than in isolation. Although our analysis draws on only two survey years (the only ones with microdata on platform workers), the patterns identified emerge consistently and clearly. Moreover, the methodological framework here implemented, which integrates ML and XAI techniques, can be readily reapplied to future waves.

### 5.1. Limitations

Notwithstanding these strengths, our findings are constrained by technical limitations inherent to the same PLUS data, which affect their robustness and generalisability. First, the data on platform workers are highly imbalanced, with the class of interest of both predictive tasks being a very small minority in both predictive tasks analysed (RQ2 and RQ3). This imbalance might have reduced the models’ ability to accurately capture the complex and varied nature of the phenomenon, thereby affecting their robustness, as indicated by the performance scores reported in B3 and B4 in S1 Appendix. Second, our cross-sectional design introduces survivorship bias: the PLUS survey captures only current platform workers, while those who have already exited remain unobserved. Our findings therefore reflect the characteristics of active platform workers and may not generalise to the full population of individuals who have ever engaged in platform labour. Moreover, the absence of a panel dimension prevents us from tracking workers over time and assessing additional labour market penalties they may experience after leaving the platform economy. Another limitation concerns the representativeness of the data on platform workers, particularly the limited inclusion of informal workers and migrants, which are notoriously under-reported in telephone surveys like PLUS. While various studies [43,55] have highlighted the significance of platform work for migrant populations, the PLUS data do not adequately reflect this reality. Indeed, only 3.8% of respondents do not possess Italian citizenship, and of these, just 1.6% come from non-European Union countries. This limitation implies that the specific dynamics of platform work among migrants are insufficiently represented in the results reported here, potentially leading to an underestimation of the extent and intensity of the phenomenon. If migrant workers, many of whom are not officially resident or lack the language skills to participate in such surveys and are therefore not captured by the PLUS sample, were fully represented, characteristics such as economic fragility and platform dependence would probably appear even more pronounced. To address this gap, future research should develop ad hoc survey tools or integrate complementary data sources, such as those from trade unions, grassroots organisations or NGOs, ensuring better coverage of the reference population.

Finally, while our analysis has focused on the socio-economic and occupational characteristics of platform workers, revealing the existence of heterogeneous labour segments and further stratification, it remains silent on the heterogeneous working conditions experienced by workers on platforms. This limitation stems in part from our emphasis on labour supply characteristics to document segmentation processes, at the expense of demand-side factors. A subsequent analysis should therefore incorporate such complementary factors (i.e., different digital platform organisational models) that shape variations in working conditions.

### 5.2. Policy implications

Despite these limitations, the analysis yields several policy-relevant implications. First, there is a need for systematic monitoring of platform work through the implementation of ad hoc surveys with a longitudinal (panel) design. Improved data collection would support a more comprehensive understanding of the phenomenon and help inform evidence-based policymaking. In particular, online tasks, as well as physical and often “invisible” activities—such as domestic and care work mediated by platforms—may be underreported or overlooked if appropriate ad hoc sampling designs are not implemented. In this respect, greater use of administrative records, including more systematic registration of platform-mediated work, could improve the measurement of these activities.

Second, the findings indicate that digital labour markets generate precarity and economic vulnerability and disproportionately rely on marginalised workers, including older or more experienced individuals facing underemployment, declining job quality, or insufficient earnings from standard employment. This points to the importance of strengthening inclusive social protection systems that cover both unemployed individuals and those in non-standard employment, in order to reduce the likelihood that vulnerable groups enter the platform economy out of necessity and remain in insecure conditions.

Third, the analysis suggests that platform workers often have caregiving responsibilities, indicating a need for greater flexibility in working time. This highlights the relevance of policies that support work–life balance across different forms of employment, facilitating the reconciliation of paid work and caregiving activities.

Fourth, platform work is frequently characterised by fragmented and unstable income sources. In this respect, policies that (i) reduce labour market flexibility by re-establishing the centrality of standard employment relationships (Italy has been one of the European economies where the shift towards flexible working arrangements and the rise in non-standard employment have been most pronounced) while strengthening protections for non-standard work; and (ii) reinforce the social protection system—particularly through income support during periods of unemployment, including for those in intermittent and non-standard jobs—could play a significant role in improving the social, economic and working conditions of this growing category of workers.

## Supporting information

S1 Appendix Additional information about the data source used, training parameters and results of the ML methods, including statistical validation. (PDF)
