# Supplementary material for: Platform workers not by chance: Exploring the digital labour markets in Italy with machine learning and explainable AI
Source: PLoS One. 2026 Jun 30;21(6):e0340237. doi: 10.1371/journal.pone.0340237 (PMC13318031; doi:10.1371/journal.pone.0340237)
Supplement: S1 Appendix — (PDF) [file pone.0340237.s001.pdf]

Platform workers not by chance: exploring the digital labour markets in Italy with machine learning and explainable AI – *S1 – Appendix*

Clara Punzi[1,2*], Valeria Cirillo[3], Dario Guarascio[4], Roberto Pellungrini[1], Fosca Giannotti[1]

1 Faculty of Science, Scuola Normale Superiore, Pisa, Italy
2 Department of Computer Science, University of Pisa, Pisa, Italy
3 Department of Political Science, University of Bari, Bari, Italy
4 Department of Economics and Law, University of Rome "La Sapienza", Rome, Italy

* Corresponding author: clara.punzi@sns.it

A Variable description

Variable	Values	RQ1-E	RQ2-U	RQ2	RQ3
**Demographics**					
Age group	18–24, 25–29, 30–39, 40–49, 50–64, 65–74	X	X	X	X
Gender	Male, Female	X	X	X	X
Marital status	Single, Married, Separated, Divorced, Widowed	X	X	X	X
Citizenship	Italian, Non-Italian	X	X	X	X
Geographical area	North-West, North-East, Center, South, Islands	X	X	X	X
City size (inhabitants)	Up to 5,000, 5,000–250,000, Over 250,000	X	X	X	X
Health status	Excellent, Good, Satisfactory, Mediocre, Poor	X	X	X	X
**Family and Household Context**					
Family type	Single, Couple with children, Couple without children, Single parent, Child of a couple, Other	X	X	X	X
Family size	1, 2, 3, 4+	X	X	X	X
Children	Yes, No	X	X	X	X
Household income	Up to €1,000, €1,001–1,500, €1,501–2,000, €2,001–3,000, Over €3,000	X	X	X	X
Household food expenditure	0–10%, 10–25%, 25–50%, 50–100%	X	X	X	X
Household mortgage expenditure	0–10%, 10–25%, 25–50%, 50–100%	X	X	X	X
Caregiving	Yes (regularly), Yes (occasionally), No	X	X	X	X
**Education**					
Personal education level	No qualification, Primary school, Middle school, High school diploma, University degree	X	X	X	X
Years since last qualification	Less than 1 year, 1–3 years, 3–5 years, 5–10 years, 10–20 years, 20+ years	X	X	X	X
Mother's education level	No qualification, Primary school, Middle school, High school diploma, University degree	X	X	X	X
Father's education level	No qualification, Primary school, Middle school, High school diploma, University degree	X	X	X	X
**Employment Status**					
Occupational status	Employed, Unemployed (seeking work), Inactive		X		
Contractual category	Not employed, Employee, Self-employed, Training contract, Other	X		X	
Occupation	Never employed, Legislators/entrepreneurs/senior managers, Intellectual/scientific/specialised, Technical, Clerical, Skilled in commerce/services, Craftsmen/skilled workers/-farmers, Machine operators/drivers, Unskilled professions, Armed forces	X	X	X	X
Work sector	Not employed, Primary, Secondary, Education, Healthcare, Tertiary	X		X	
Part-time status	Not employed, Incompatible contract, Yes (voluntary), Yes (involuntary), No, Don't know	X		X	
Side job	Contract not compatible, Yes, No	X		X	
Weekly working hours	Not employed, Less than 16, 17–20, 21–30, 31–36, 37–40, 41–50, 51+	X		X	

Continued on next page

Variable	Values	RQ1-E	RQ2-U	RQ2	RQ3
Monthly net income	Not compatible (self-employed), Less than €1,000, €1,001–1,500, €1,501–2,000, Over €2,001	X		X	
Skill mismatch	Not employed, Much higher, Slightly higher, About the same, Slightly lower, Much lower	X		X	
Employment history	Currently employed, Yes (previously employed), No (never employed before)		X		
Years since first job	Not employed, Less than 1 year, 1–3 years, 3–5 years, 5–10 years, 10–20 years, 20+ years	X		X	
**Economic vulnerabilities**					
Maximum extraordinary expense	None, Less than €300, €300–800, €800–2,000, Over €2,000	X	X	X	X
Postponement of medical treatment	Yes, No	X	X	X	X
Income support	Yes (currently), Yes but no longer, No never, No not compatible	X		X	
**Digital platforms**					
Relevance of platform work	Does not work on platforms, Essential to meet basic needs, Important but not essential, Convenient but not necessary	X	X		
Type of platform work	Does not work online, Parcel delivery, Food delivery, Online tasks (data entry, software etc.), Household services (cleaning plumbing etc.), Passenger transport, Other	X	X		
Online sales	Yes, No	X	X		
Online home-sharing	Yes, No	X	X		
**Survey year**					
Year	2018, 2021	X	X	X	X

Table A1. Variable description. Overview of processed variables derived from the INAPP PLUS survey, listing possible values (second column) and their inclusion across research questions RQ1–RQ3 (last four columns).

B Classification model training

For both RQ2 and RQ3, the binary classification models were first optimized through hyperparameter tuning, followed by model selection using critical difference diagrams.

Hyperparameter optimization Hyperparameters were tuned via 5-fold cross-validation on the training set, with the best configuration chosen based on the average F_1 score across folds. A grid search strategy was employed to explore the hyperparameter space. When supported by the underlying classifier, sample weights and a validation set were additionally incorporated to refine the tuning process. The complete list of hyperparameters and their candidate values for each black-box model is reported in Table B2.

Model performance The final model for each research question was determined using 30-fold cross-validation. Since the binary classification task was highly imbalanced in both cases, multiple metrics were taken into consideration, namely accuracy, precision, recall, F_1-score and ROC-AUC score. Then, the model that maximised the majority of performance metrics was chosen. To confirm the results, we computed the relative performance of multiple classifiers evaluated on all metrics and verified their statistical significant difference via pairwise tests using a one-sided Wilcoxon signed-rank test with the Holm correction. The classifiers marked by solid horizontal lines in the resulting critical difference diagrams represent groups showing no significant difference in performance.

Table B2. Hyperparameter tuning.

Model	Hyperparameter	Hyperparameter values	Fit parameters
Logit	penalty<br>C<br>max iterations<br>solver	L1, L2, elasticnet, None<br>0.01, 0.1, 1, 10<br>100, 500<br>lbfgs, liblinear, saga	sample weights
Decision Tree	max depth<br>min samples split<br>min samples leaf<br>max features	None, 1, 3, 5, 10<br>2, 5, 10<br>1, 2, 4<br>sqrt, log2	sample weights
Random Forest	num estimators<br>min samples split<br>min samples leaf<br>bootstrap	50, 100, 200, 300<br>2, 5, 10<br>1, 2, 4<br>True, False	sample weights
MLP	num hidden layers<br>activation<br>solver<br>learning rate<br>alpha	(50,), (100,), (50, 50), (100, 100)<br>tanh, relu<br>lbfgs, adam<br>constant, adaptive<br>0.0001, 0.05	sample weights
XGBoost	num estimators<br>max depth<br>learning rate<br>subsample	50, 100, 200, 300<br>3, 6, 9<br>0.001, 0.01, 0.1, 1<br>0.5, 0.7, 1	sample weights<br>evaluation set
CatBoost	num estimators<br>max depth<br>learning rate	100, 200, 300<br>3, 6, 9<br>0.001, 0.01, 0.1, 1	sample weights<br>evaluation set
TabNet	width of the decision prediction layer<br>width of the attention embedding<br>learning rate of the optimizer	8, 16, 24<br>8, 16, 24<br>0.015, 0.02, 0.025	sample weights<br>evaluation set

Hyperparameter configurations for all trained models in RQ2 and RQ3. The best hyperparameters found by GridSearch to maximize the F_1-score are highlighted in red for RQ2 and, if differing, in blue for RQ3.

Overall, CatBoost, XGBoost and Random Forest demonstrated good performance, often without substantial differences. The CatBoost classifier was selected for RQ2 since it exhibited greater performance (but without statistical significance; see Figure B1, left column) across all metrics, except for recall, where it ranked second (see Table B3). Similarly, as for RQ3, the XGBoost model was chosen among the top scoring due to the absence of statistically significant differences when compared to comparable models (see Figure B1, right columns), while also demonstrating shorter training and fitting time (see Table B4).

Table B3. Performance scores of RQ2 classification models.

Model	Fit time	Score time	Accuracy	Precision	Recall	F_1	ROC-AUC
Logit	0.301	0.080	0.980	0.930	0.602	0.717	0.895
Decision Tree	0.089	0.072	0.975	0.792	0.634	0.693	0.874
Random Forest	2.200	0.112	0.983	0.952	0.666	0.769	0.942
CatBoost	5.325	0.113	**0.986**	**0.953**	0.719	**0.811**	**0.943**
XGBoost	0.645	0.093	0.985	0.914	**0.738**	0.807	0.935
MLP	5.581	0.094	0.985	0.936	0.712	0.799	0.921
TabNet	201.926	0.086	0.983	0.824	0.662	0.722	0.868

Performance of all candidate models in RQ2 was evaluated through 30-fold cross-validation. The row highlighted in green corresponds to the final selection, which optimises the majority of performance metrics.

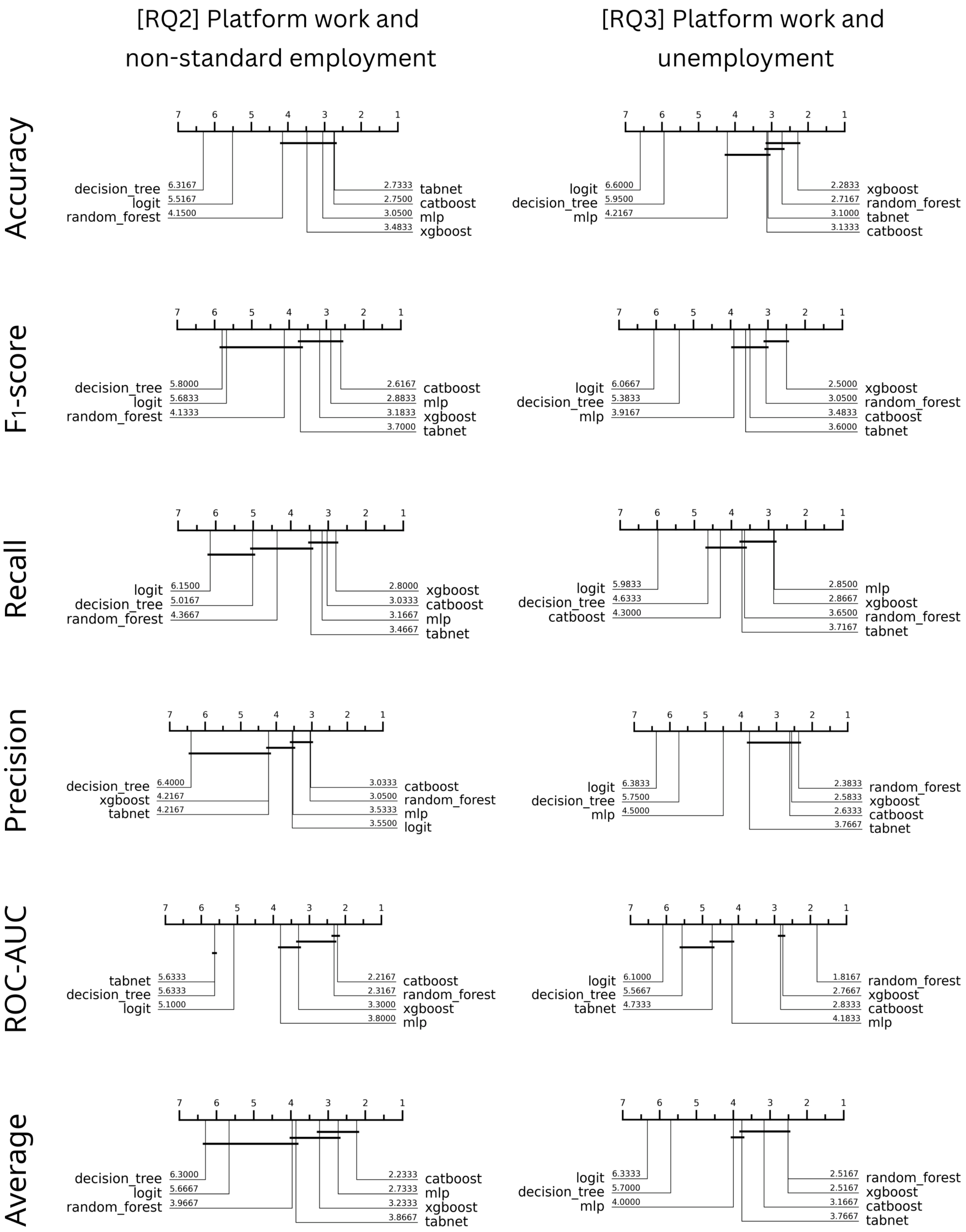

Fig B1. Critical difference diagrams of all models trained for RQ2 and RQ3, computed with respect to multiple metrics. The underlying data correspond to the performance scores obtained through 30-fold cross-validation

Table B4. Performance scores of RQ3 classification models.

Model	Fit time	Score time	Accuracy	Precision	Recall	F_1	ROC-AUC
Logit	2.461	0.109	0.938	0.198	0.152	0.154	0.672
Decision Tree	0.047	0.057	0.964	0.486	0.588	0.524	0.819
Random Forest	3.457	0.148	0.985	0.851	0.624	0.669	**0.930**
CatBoost	5.266	0.086	0.983	**0.870**	0.583	0.650	0.916
XGBoost	**0.886**	0.083	**0.986**	0.841	**0.662**	**0.698**	0.899
MLP	17.284	0.073	0.975	0.625	0.636	0.606	0.877
TabNet	122.921	0.059	0.960	0.230	0.123	0.145	0.747

Performance of all candidate models in RQ3 evaluated through 30-fold cross-validation. The row highlighted in green corresponds to the final selection, which optimises the majority of performance metrics.

C Additional results

C.1 RQ1: Cluster description

	Sheltered youth			Accumulationists			Vulnerable jobseekers			Precarious workforce		
	mode	mean	std	mode	mean	std	mode	mean	std	mode	mean	std
City size	(2) 5-250 K	2.06	0.56	(2) 5-250 K	2.08	0.62	(2) 5-250 K	2.02	0.68	(2) 5-250 K	1.83	0.72
Geographical area	(4) South	3.05	1.37	(1) North-West	2.54	1.37	(4) South	2.83	1.42	(4) South	3.34	1.33
Gender	(1) Male	1.43	0.49	(1) Male	1.45	0.50	(1) Male	1.41	0.49	(1) Male	1.19	0.39
Age group	(2) 25-29	1.76	0.72	(2) 25-29	2.75	1.09	(3) 30-39	3.41	1.15	(4) 40-49	3.77	1.02
Occupational status	(3) Inactive	2.52	0.50	(1) Employed	1.00	0.00	(2) Job seeker	2.37	0.48	(1) Employed	1.00	0.00
Family type	(5) Child of a couple	4.65	1.47	(5) Child of a couple	3.40	1.75	(2) Couple with children	2.65	1.61	(2) Couple with children	2.09	1.15
Family size	(4) 4+	3.14	0.94	(4) 4+	2.72	1.06	(4) 4+	2.67	1.14	(1) 1	2.50	1.17
Children	(0) No	0.03	0.17	(0) No	0.23	0.42	(1) Yes	0.64	0.48	(1) Yes	0.88	0.32
Citizenship	(1) Italian	1.03	0.17	(1) Italian	1.04	0.19	(1) Italian	1.05	0.22	(1) Italian	1.00	0.07
Marital status	(1) Single	1.09	0.34	(1) Single	1.32	0.52	(2) Couple	1.69	0.64	(2) Couple	1.60	0.57
Own education level	(3) High school	3.27	0.56	(3) High school	3.39	0.58	(3) High school	2.81	0.63	(3) High school	3.36	0.56
Father's education level	(3) High school	2.87	0.94	(3) High school	2.59	0.94	(3) High school	2.44	0.99	(3) High school	2.97	0.91
Mother's educational	(3) High school	2.88	0.87	(3) High school	2.64	0.93	(3) High school	2.37	0.96	(3) High school	3.04	0.87
Years since last qualification	(3) 5-10 years	2.05	1.31	(4) 10-20 years	3.15	1.45	(5) 20+ years	4.06	1.29	(5) 20+ years	4.14	1.11
Years since first job	(-1) Not employed	-1.00	0.00	(4) 10-20 years	2.98	1.51	(-1) Not employed	-1.00	0.00	(5) 20+ years	3.91	1.41
Weekly working hours	(-1) Not employed	-1.00	0.00	(0) <16 hours	0.78	0.82	(-1) Not employed	-1.00	0.00	(0) <16 hours	0.16	0.46
Occupation (also previous)	(-1) Never employed	2.47	3.09	(2) Highly specialised professions	3.78	1.85	(4) Office work	3.38	2.69	(4) Office work	4.09	1.82
Work sector	(-1) Not employed	-1.00	0.00	(5) Tertiary	4.05	1.55	(-1) Not employed	-1.00	0.00	(5) Tertiary	3.71	1.51
Contractual category	(-1) Not employed	-1.00	0.00	(2) Self-employed	1.43	0.63	(-1) Not employed	-1.00	0.00	(1) Employee	1.38	0.72
Part-time status	(-1) Not employed	-1.00	0.00	(3) No	1.92	1.31	(-1) Not employed	-1.00	0.00	(1) Yes, voluntary	1.60	1.07
Side job	(-1) Contract not compatible	-1.00	0.00	(-1) Contract not compatible	-0.37	0.75	(-1) Contract not compatible	-1.00	0.00	(1) Yes	0.34	0.88
Income support	(0) No	0.00	0.00	(0) No	0.12	0.33	(0) No	0.00	0.00	(0) No	0.23	0.42
Skill mismatch	(-1) Not employed	-1.00	0.00	(3) More or less the same	2.26	1.01	(-1) Not employed	-1.00	0.00	(2) Slightly higher	2.21	0.70
Monthly net income	(-1) Non compatible (unemployed)	-1.00	0.00	(-1) Non compatible (self-employed)	-0.40	0.89	(-1) Non compatible (unemployed)	-1.00	0.00	(0) Less than €1.000	0.47	1.39
Monthly household mortgage expenditure	(1) 0-10%	1.82	0.90	(1) 0-10%	2.03	0.93	(1) 0-10%	2.02	0.95	(2) 10-25%	2.19	0.78
Monthly household food expenditure	(3) 0-10%	2.42	0.70	(3) 0-10%	2.49	0.74	(3) 0-10%	2.59	0.71	(2) 10-25%	2.38	0.58
Max sudden expense	(2) €300-800	1.83	1.12	(2) €300-800	2.48	1.23	(1) <€300	1.77	1.16	(2) €300-800	2.45	0.80
Postponement of medical treatment	(0) No	0.26	0.44	(0) No	0.23	0.42	(1) Yes	0.73	0.44	(1) Yes	0.88	0.33
Health status	(2) Good	1.91	0.76	(2) Good	2.09	0.83	(2) Good	2.48	0.86	(2) Good	2.27	0.78
Caregiving	(3) No	2.72	0.59	(3) No	2.59	0.72	(1) Yes, regularly	1.89	0.83	(1) Yes, regularly	1.37	0.54
Household income	(3) €1.5-2K	3.08	1.30	(4) €2-3K	3.39	1.22	(2) €1-1.5K	2.43	1.12	(3) €1.5-2K	3.06	1.09
Online sale	(0) No	0.46	0.50	(0) No	0.34	0.47	(1) Yes	0.57	0.50	(1) Yes	0.77	0.42
Online home-sharing	(0) No	0.07	0.25	(0) No	0.07	0.25	(0)No	0.41	0.49	(1) Yes	0.75	0.43
Relevance of platform labour	(3) Convenient but not necessary	2.30	0.75	(3) Convenient but not necessary	2.27	0.78	(2) Important but not essential	1.94	0.73	(1) Essential to meet basic need	1.79	0.63
Task on platform	(2) Food delivery	3.17	1.71	(3) Online tasks	3.14	1.68	(3) Online tasks	3.18	1.53	(2) Food delivery	2.36	0.89

Table C5. Cluster description. The table reports the representative values for each variable across clusters (including modes, means and standard deviations), providing detailed descriptive statistics about the four digital platform labour profiles.

C.2 RQ2 & RQ3: Feature importance

In order to determine the factors influencing the transition to digital platform work in both RQ2 and RQ3, we derived the ranking of feature importance scores. These were assessed and contrasted using several methodologies: 1) the built-in functions of the classifiers used, namely CatBoost and XGBoost, 2) standard SHAP values and 3) StableSHAP , a novel technique that enhances SHAP via a sampling procedure that finds the top-K most important features, possibly with their relative ordering, with high probability guarantees. Given that StableSHAP currently yields only local explanations (i.e., stable feature rankings relative to individual instances), we developed a strategy to derive global feature rankings for comparative analysis with other methods: we calculated local StableSHAP values for a subset of test instances (in a number balancing method performance and speed) and subsequently estimated the corresponding critical difference diagram encompassing all features.

RQ2 In RQ2, we picked a 10% subset of inputs from the test set, stratified by the target variable. For each data point, we ran StableSHAP to identify the ordered sets of the top-5 and top-10 features, ensuring their probability of correctness (including their relative ordering) is at least $1-\alpha$, where $\alpha = 0.1$. After that, we built critical difference diagrams (Fig C3) based on the ranking of all features determined by the importance scores generated from StableSHAP for all samples in the subset where the algorithm converged, in order to assess the statistical significance of the global ranking. Of the 320 samples in the chosen subset, StableSHAP converged on 195 instances in the top-5 method and 54 in the top-10. Considering the hardness and lengthy execution time of the method with big K, together with its consistency with the top-5 results already emerging from a limited sample set, we will focus on the latter for the following analyses. Notably, the ranking suggested by the built-in CatBoost method (Fig C2) and by SHAP (Fig 3) was largely corroborated by the statistical tests in StableSHAP. Therefore, the main body of the paper only presents the results pertaining to SHAP, which allows a deeper examination of feature contributions. Fig C7 displays the dependence scatter plots of the most relevant features, which show the effect of each single feature on the predictions made by the classification model. In the plots, each point represents an individual's SHAP value for that feature, with values above zero (dashed line) pushing toward platform worker and values below zero pushing toward other forms of non-standard worker. The underlying grey bars show the distribution of respondents across categories.

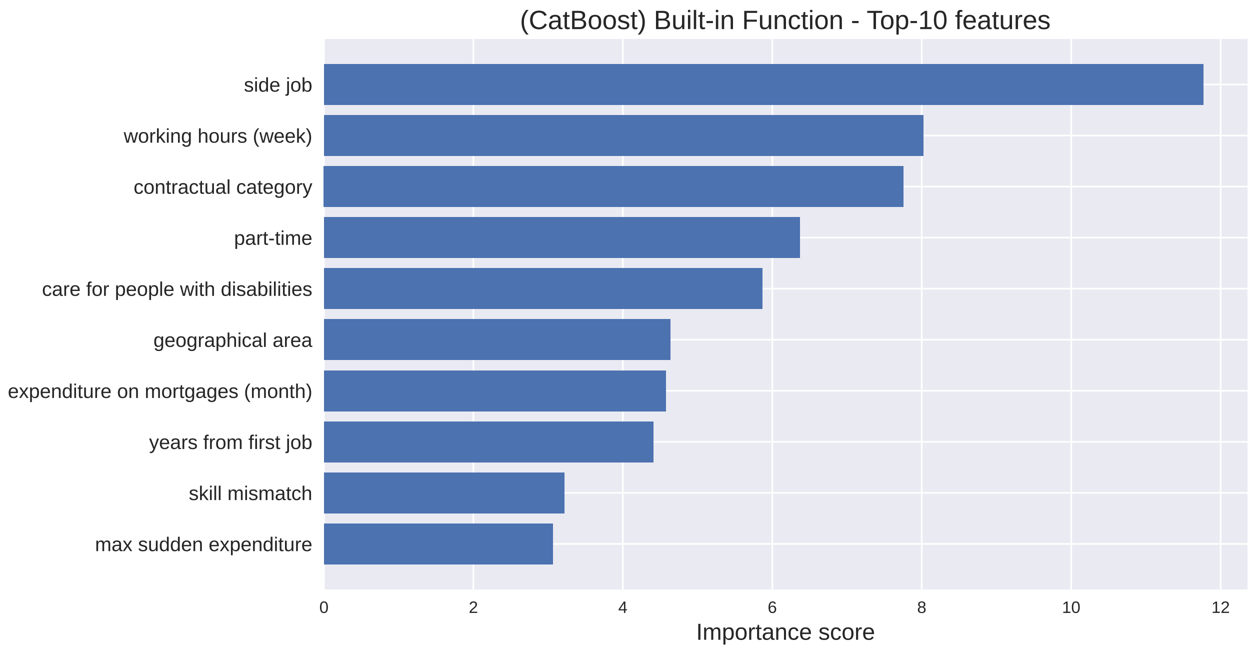

Fig C2. CatBoost feature importance ranking. Top-10 factors predicting engagement with the digital platform workforce among non-standard workers (RQ2), computed through the CatBoost built-in function.

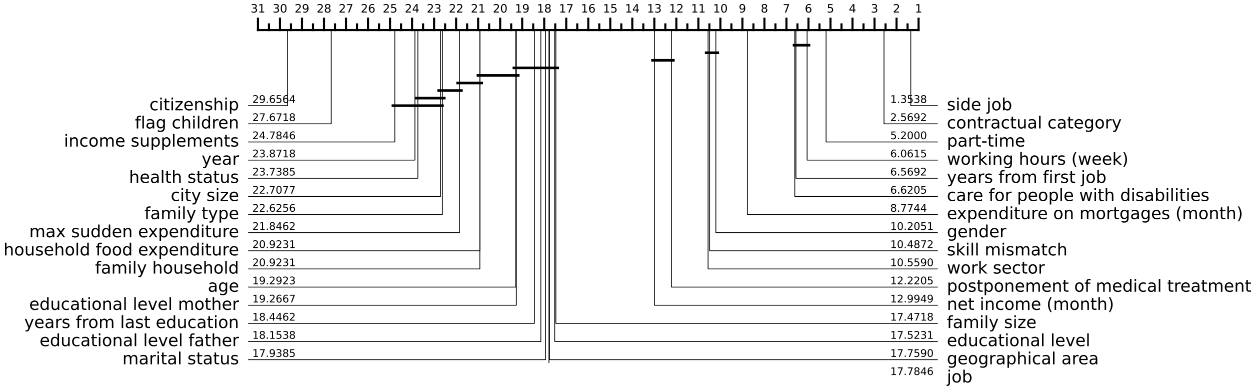

Fig C3. Critical difference diagram of all the feature importance scores in RQ2. The underlying data correspond to the importance scores of the converged samples of (top-5) StableSHAP, computed on 10% of the test set.

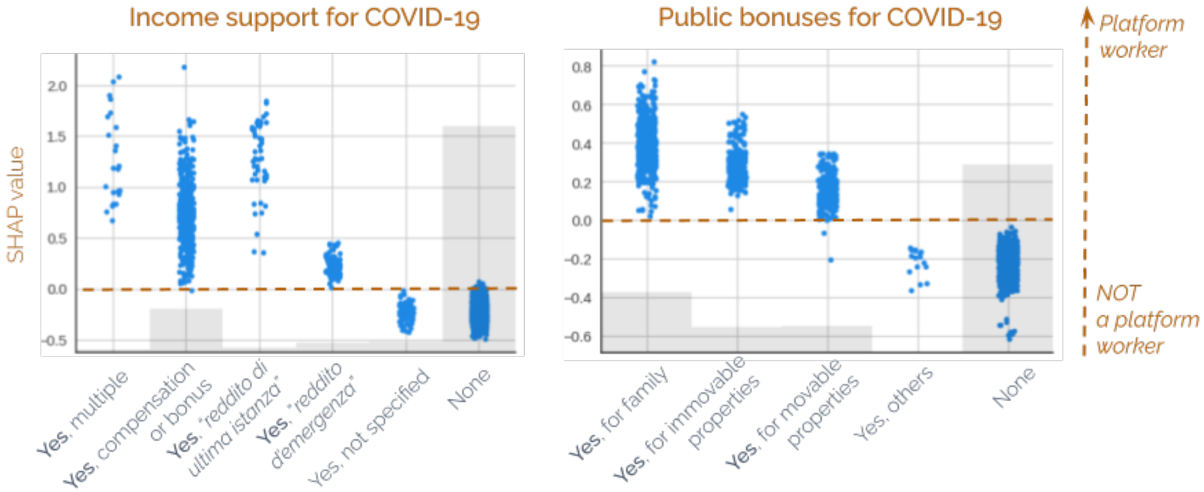

Fig C4. Contribution of pandemic-related variables. SHAP scatter plots showcasing key factors identified as positive predictors of engagement with the digital platform in RQ2, restricted to the sampling year 2021. Each point represents an individual's SHAP value for that feature, with values above zero (dashed line) pushing toward platform workers (bold categories) and values below zero pushing toward other forms of non-standard workers. The underlying grey bars show the distribution of respondents across categories.

RQ3 Concerning RQ3, the calculation of stable SHAP values using StableSHAP proved to be harder, as the algorithm converged on a minimal fraction of the chosen 10% of the test set (9 occurrences out of 221). Hence, we relied on the weaker version of the method that evaluates whether the set of K largest observed elements is guaranteed to have the highest means with probability exceeding 1α, $\alpha = 0.1$, whilst disregarding the order inside the top-K. We calculated the unordered top-5 stable features from 20% of the test set, identifying 117 convergent samples, and illustrated the entire ranking through the critical difference plot in Fig C6. However, the results did not demonstrate significant differentiation in feature ranking, as indicated by the large cliques in the plots.

In contrast to the study conducted on RQ2, the feature importance ranking for RQ3 displayed much less consistency among techniques, namely, XGBoost built-in function (Fig C5), SHAP, and StableSHAP(Fig C6). Nonetheless, disregarding the order, a few top-scoring features can be identified. For a more fine-grained analysis, Fig C8 displays the dependence scatter plots of the most relevant features, which show the effect of each single feature on the predictions made by the classification model.

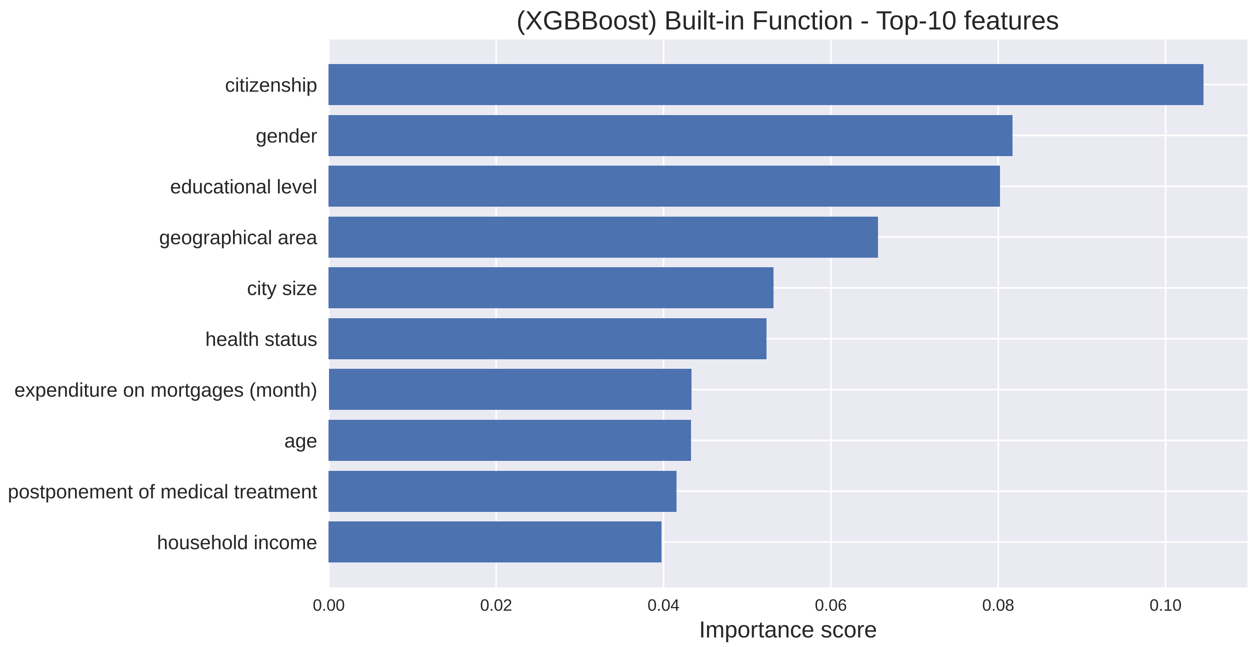

Fig C5. XGBBoost feature importance ranking. Top-10 factors predicting engagement with the digital platform workforce among job-seekers below 50 years old (RQ3), computed through the XGBBoost built-in function.

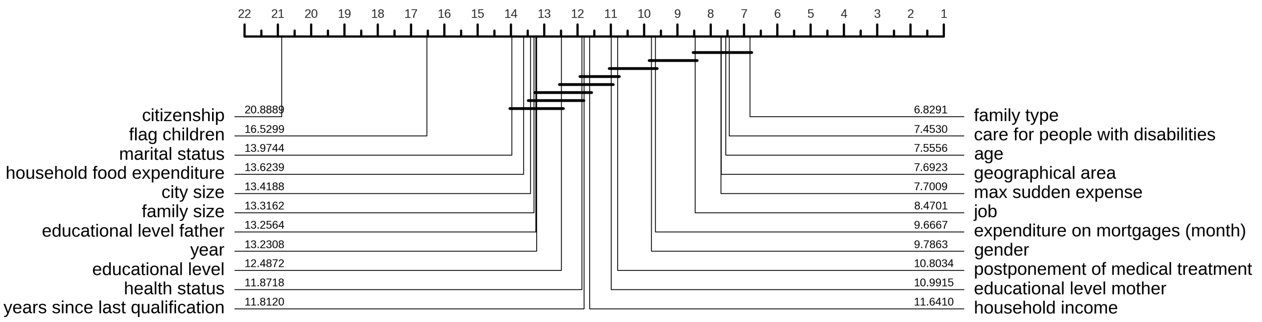

Fig C6. Critical difference diagram of all the feature importance scores in RQ3. The underlying data correspond to the importance scores of the converged samples of (unordered) top-5 StableSHAP, computed on 20% of the test set.

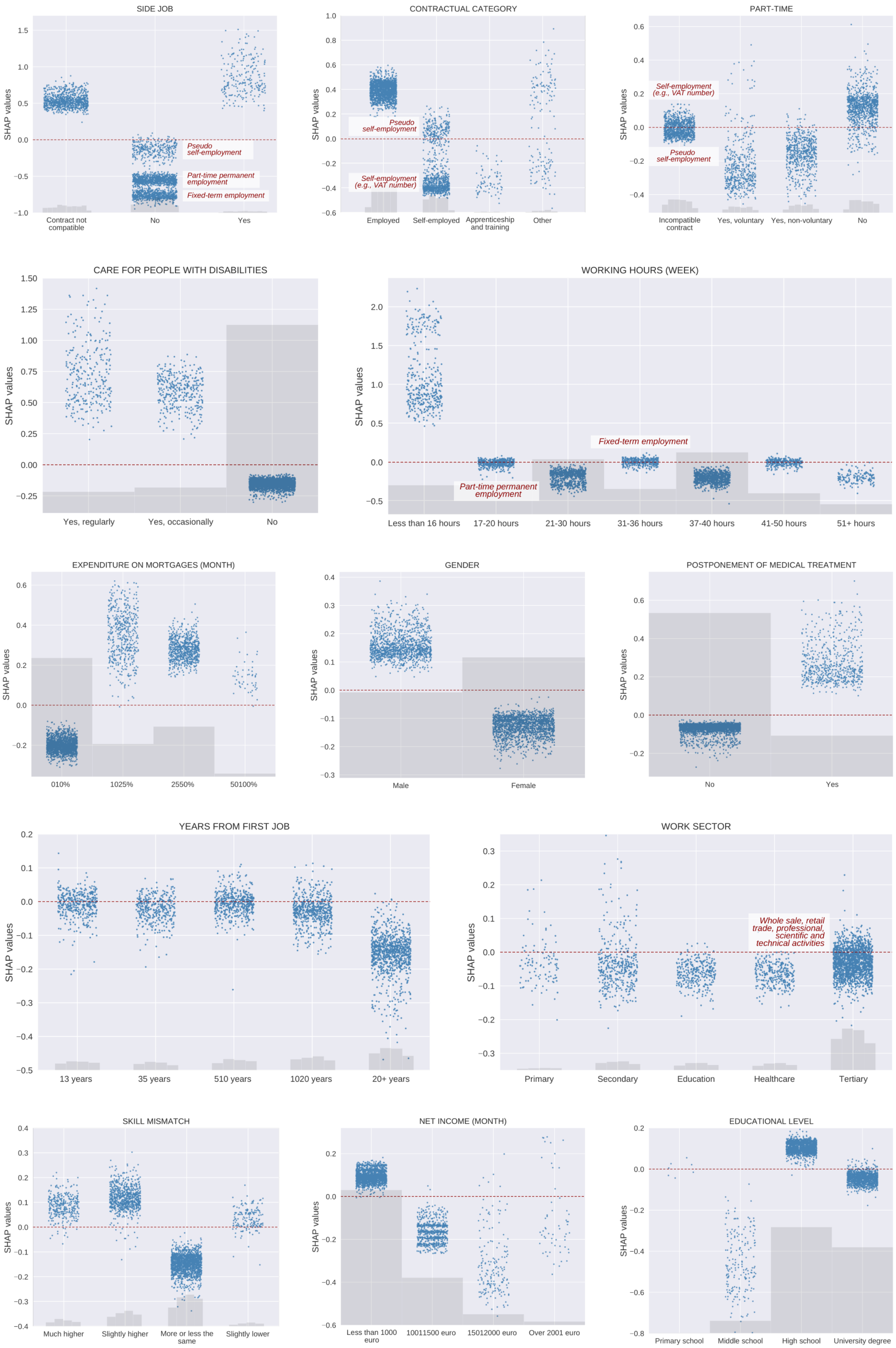

Fig C7. Dependence scatter plots showing the effect of each of the top features on the predictions made by the RQ2 classification model.

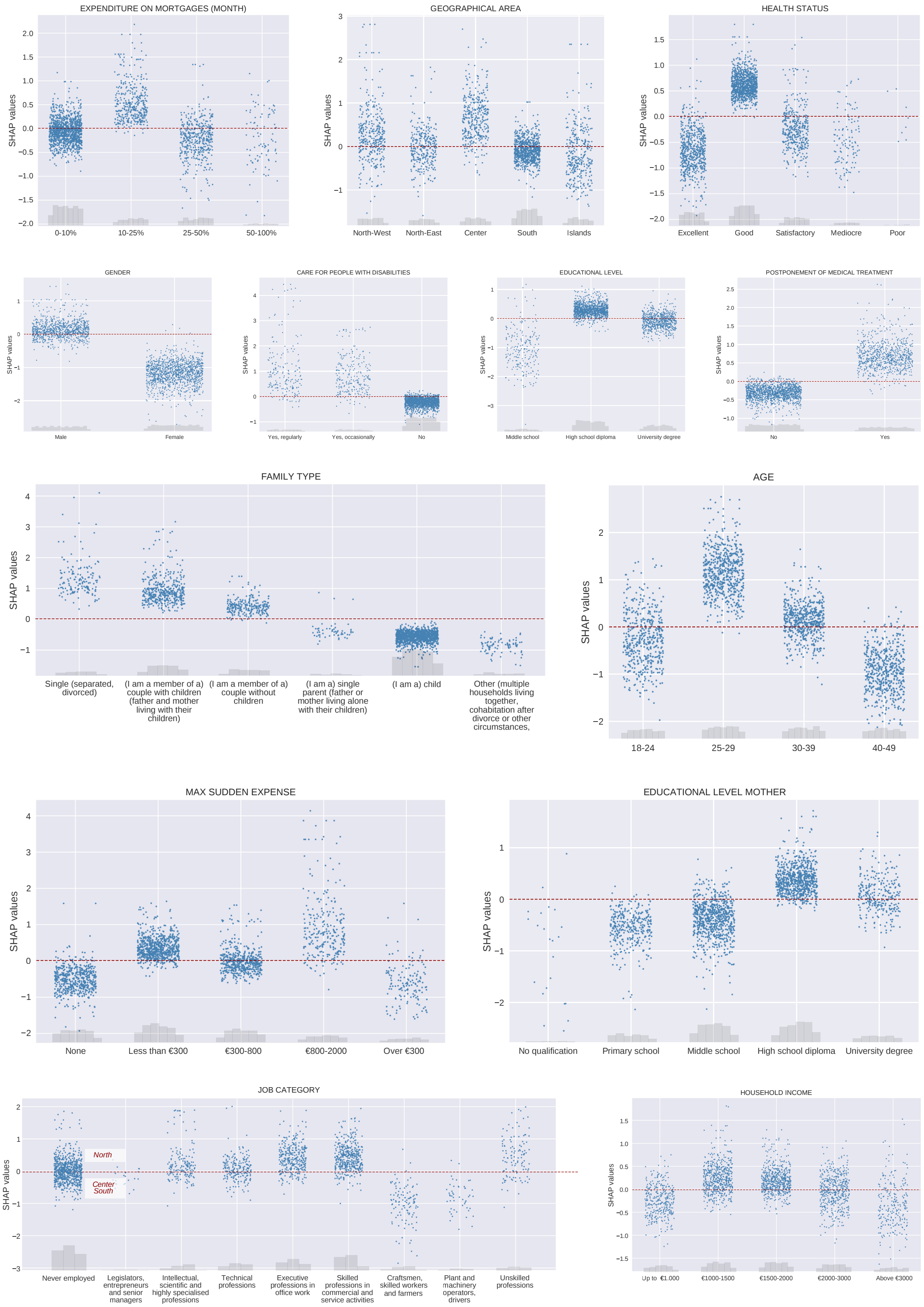

Fig C8. Dependence scatter plots showing the effect of each of the top features on the predictions made by the RQ3 classification model.
