# Supplementary material for: Platform workers not by chance: Exploring the digital labour markets in Italy with machine learning and explainable AI
Source: PLoS One. 2026 Jun 30;21(6):e0340237. doi: 10.1371/journal.pone.0340237 (PMC13318031; doi:10.1371/journal.pone.0340237)
Supplement: Attachment — Submitted filename: Response to reviewers.pdf [file pone.0340237.s002.pdf]

Comment of the Editor *[from the "Decision Letter" to PONE-D-25-60832]*

"We have noted that you have used the 2021 wave of the INAPP-PLUS survey and stated that this is the latest version - however, we have found that results from the 2022, and 2023 wave have been reported. The usage of an older dataset may not be able to make up-to-date conclusions. Further, the usage of an old dataset could also imply that the policy recommendations in the manuscript may no longer be appropriate or applied to the current economic scenario."

Response from the Authors

Dear Editor,

Thank you for your evaluation of our manuscript and for your interest in the topics addressed. We fully share the importance of relying on the most recent and reliable data available. However, the rejection appears to stem from a misunderstanding regarding the release dates of INAPP PLUS survey data. With this letter, we would like to further clarify and provide evidences that the two most recent waves of this survey containing valid and reliable information on platform workers are indeed 2018 and 2021.

First of all, although a 2022 wave of INAPP PLUS exists, the Institute's Statistical Office explicitly informed us that the module on the gig economy for that year was not disseminated due to severe data quality issues (primarily the extremely low number of observations). For this reason, no platform work data from 2022 are available for research use. Moreover, no PLUS 2023 data have been collected nor released at all. Should the editorial team have access to different information, we would be grateful to be informed so that we may verify it with the relevant INAPP contacts.

We can support these statements with multiple official INAPP sources:

- The most recent INAPP PLUS report (the official publication through which the data are released once finalised) shows that the latest available wave is 2022, that no 2023 data exist, and that the 2022 release does not includ data on platform work for the reasons mentioned above.
- The INAPP webpage dedicated to platform workers also reports 2021 as the most recent wave containing platform work data, which is precisely the dataset we employed.
- A newly published INAPP report on the platform economy similarly indicates that the latest evidence on platform work from PLUS refers to 2021: *"The most recent data on the extent of the phenomenon in Italy have been released by INAPP and Istat, thanks to the forms included in the 2021 Plus Survey (Bergamante et al. 2022) and in the 2022 Labour Force Survey (Istat 2024)."* (p. 52; translation by the authors).

Finally, we note that two of the co-authors (Dario Guarascio and Valeria Cirillo) were INAPP researchers until 2020 and were directly involved in the working group that designed and introduced in PLUS the module on the gig economy. They can therefore personally confirm the accuracy of the information reported above.

We fully appreciate PLOS ONE’s commitment to methodological rigor and to the use of up-to-date data. For the reasons documented here, we respectfully submit that our manuscript does indeed rely on the most recent and reliable PLUS data currently available for the study of platform workers in Italy.

We have also addressed all of these elements in the body of the article. Specifically, we have clarified the relevance and timing of the utilised data in the methodology (lines 145-147, 166-169) and conclusion (493-501) sections.

We remain at your disposal for any further clarification or supporting documentation.

Thank you very much for your attention.

Kind regards,

Clara Punzi, Valeria Cirillo, Dario Guarascio, Roberto Pellungrini, Fosca Giannotti
