# Supplementary material for: Platform workers not by chance: Exploring the digital labour markets in Italy with machine learning and explainable AI
Source: PLoS One. 2026 Jun 30;21(6):e0340237. doi: 10.1371/journal.pone.0340237 (PMC13318031; doi:10.1371/journal.pone.0340237)
Supplement: Attachment — Submitted filename: Response to reviewers_v2.pdf [file pone.0340237.s004.pdf]

Dear Editor,

We sincerely thank you for the opportunity to revise and resubmit our manuscript.

We have carefully addressed all the valuable suggestions provided by the reviewers, which we believe have substantially improved the clarity and overall effectiveness of our contribution. In particular, we have strengthened both the methodology and the interpretation of the results, and we have better framed our analysis in relation to the existing literature on platform and non-standard work.

In the following, we provide detailed responses to each of the reviewers' comments. All revisions and additions are highlighted in red in the main text.

Sincerely yours,

Reviewer #1:

Overall observations

(1) The manuscript relies on a broad and somewhat implicit definition of platform work. While this aligns with the PLUS survey structure, the analytical consequences of this definition are not sufficiently discussed. The distinction between primary vs. secondary platform income is central to your argument, yet it is not clearly operationalised upfront. Different forms of platform work (location-based vs web-based; task-based vs continuous) are mentioned theoretically but not analytically differentiated. It does matter because without conceptual tightening, readers may question whether the observed heterogeneity reflects: real labour-market stratification, or definitional breadth and measurement noise.
Therefore, authors can add a short subsection in Materials and Methods explicitly clarifying how PLUS defines platform work; what types of activities are included/excluded; how dependence on platform income is measured. Explicitly acknowledge that heterogeneity may partly stem from definitional aggregation and explain why this does not invalidate the findings.

RESPONSE: Thank you for this thoughtful comment. In response, we have made the following additions to the "Data" section. First, we have explicitly added the definition of platform work as operationalised by the PLUS survey, including the exact wording of the screening question (same for 2018 and 2021). Second, we now clearly state that the limited sample size prevents differentiated analyses across platform task types (e.g., delivery vs. online tasks) or by primary versus secondary platform income. We explain that this aggregation does not invalidate our findings, as different task types share common structural features, such as precarious conditions, economic vulnerability, and limited protections. Third, while being transparent about these limitations, we have added descriptive statistics on platform tasks and occupational status

providing an additional and insightful piece of information concerning the characteristics of platform work in Italy.

(2) While ML and XAI are carefully implemented, the manuscript does not sufficiently justify why ML is necessary for the research questions posed. Many of the conclusions (e.g., vulnerability, age gradients, regional divides) could plausibly emerge from logistic regression or multinomial models. The paper emphasises “non-linearities” and “complex interactions” but rarely demonstrates what ML reveals that standard models would miss. It is important to clear because readers often require a clear methodological added value, not just technical sophistication. So, authors can add a benchmark comparison (even in an appendix): e.g., logistic regression vs CatBoost/XGBoost for RQ2 and RQ3., and explicitly state: which relationships are non-linear and which interactions would be difficult to pre-specify in traditional models.

RESPONSE: We thank the reviewer for this important comment. To clarify the methodological contribution of the paper, we have now included a benchmark comparison with logistic regression models for both RQ2 and RQ3 (reported in Appendix B, Table 4 and 5 - red rows). The results show that logistic regression consistently underperforms relative to the selected ML models (CatBoost and XGBoost) across all evaluation metrics, suggesting that the relationships underlying platform work participation are not fully captured by linear specifications. This supports our initial motivation for employing ML methods, namely their ability to flexibly capture non-linear patterns and higher-order interactions without requiring strong a priori assumptions. We have revised the methodology section to make this justification more explicit and better highlight our methodological contribution.

(3) In several places, SHAP-based importance rankings are discussed in language that implies causality or behavioural motivation (e.g., “factors lead jobseekers to turn to platform work”).
Authors can consider the overestimation wording; for example, replace “leads to,” “drives,” “is strongly associated with,” “is predictive of,” or “contributes to the model’s classification.” Also can add a clear methodological disclaimer early in the Results section explaining: SHAP ≠ causal inference.

RESPONSE: Thank you for this important observation. We have: (1) added a methodological disclaimer in the “Explainable AI models” section clarifying that SHAP values do not imply causal relationships, and (2) revised the “Results” and “Discussion” section to replace causal language with strictly correlational phrasing, trying not to overload the reader with repetitions of terminology. All changes are highlighted in red.

(4) The manuscript frequently references COVID-19 as an amplifier of platform work dynamics, but: the analytical strategy for comparing 2018 vs 2021 is not fully explicit; it remains unclear whether observed differences reflect pandemic effects or compositional changes.
Could you please clarify whether: models were pooled with year dummies, or estimated separately by year. Include at least one interaction or stratified analysis by year to support pandemic-specific claims. Alternatively, soften claims and frame COVID-19 as a contextual backdrop rather than an identified causal shock.

RESPONSE: Thank you for this observation. We have clarified in the text the analytical strategy for comparing 2018 vs 2021: as limited sample size prevented separate year-specific models, we included year as a binary feature in a pooled model and performed SHAP cohort analysis accordingly. We have also softened all causal framing, adding references to the existing literature where appropriate.

(5) The PLUS survey is strong, but platform work is notoriously under-reported in telephone surveys. So you can add a dedicated Limitations subsection addressing under-coverage of informal or migrant platform workers; recall bias and misclassification; survivorship bias (those who exited platform work are invisible); implications of survey weights in ML contexts. This will substantially strengthen the paper's credibility.

RESPONSE: Thank you for these constructive suggestions. Please note that most of the concerns raised were already addressed in the section now renamed "Limitations" (formerly "Conclusion"). In response to your comment, we have additionally: (1) added a specific reference to survivorship bias; (2) explicitly mentioned informal workers alongside migrants when discussing under-coverage; (3) clarified that misclassification measures are reported in the Appendix. Also note that survey weights were applied in all classification models, as specified in the Methods section.

Section-specific comments

1) Abstract

Slightly reduce normative language ("extractive tendencies"). Clarify that findings are associational and predictive.

RESPONSE: We thank the Reviewer for raising this point. We have reduced the use of normative language and clarified the non-causal nature of both the analysis and the results.

2) Introduction

Authors did an excellent synthesis of labour sociology and platform economy literature with strong contextualisation of Italy. Yet explicitly link each theoretical strand to a variable or modelling choice. RQ3 implicitly suggests causal pathways ("which factors lead jobseekers…"). Rephrase RQ3 to emphasise probabilistic association, not causation.

RESPONSE: We thank the Reviewer for raising this point. In response, we have added a new paragraph in the subsection 'Scope and Structure of the Study' that explicitly links the groups of variables used in the analysis to the relevant literature. Moreover, we have rephrased RQ3 to remove causal language.

3) Methods

Preprocessing steps are dense and hard to follow for non-ML readers. Add a schematic workflow figure. Move some technical detail to an appendix.

RESPONSE: Thank you for this helpful suggestion. In response, we have added a schematic workflow figure (now Figure 1) that visually summarises the main steps of our explainable machine learning pipeline, making the overall methodology more accessible to non-ML readers. However, we have retained the detailed preprocessing descriptions in the main text, as another reviewer specifically requested more technical detail.

4) Results
Some repetition across RQ1 and RQ2. Cluster labels (e.g., "accumulationists," "sheltered youth") are evocative but subjective. However, Justify cluster naming more explicitly. Add a concise summary table of cluster characteristics.

RESPONSE: We thank the reviewer for this comment. To enhance the reader's understanding of the proposed terminology for the clusters, we have included a concise summary table of cluster characteristics (Table 2, red background), as well as a comprehensive set of descriptive statistics, encompassing modes, means, and standard deviations for all variables by cluster in Table 7 (red background) of the Appendix.

5) Discussion and conclusions
Normative tone occasionally exceeds evidence. Policy implications could be more concrete. Authors could separate empirical conclusions from normative reflections. Add 2–3 clearly specified policy implications (e.g., social protection design, labour statistics).

RESPONSE: We thank the reviewer for raising this point. We have revised the conclusions including policy implications and removing normative tone.

Reviewer #2:

Major comments

1. Positioning within the literature
While the manuscript reviews a number of studies on platform work, the specific contribution of the paper relative to the existing literature could be articulated more clearly.
In particular, it would be useful to clarify the empirical or methodological gap the study aims to fill and how the findings extend or challenge previous research on platform work and labour market segmentation;
The paper suggests that platform work is not predominantly a youth phenomenon but is instead associated with economically vulnerable workers. This is an interesting claim, but it should be discussed more systematically in relation to the existing literature on labour market dualism, precarious employment, and gig work.

RESPONSE: We thank the reviewer for raising this point. We have rewritten the introduction to explicitly refer to contributions on labour market dualism in order to better frame our analysis.

2. Justification for the use of machine learning

The manuscript relies on machine learning techniques combined with explainable AI tools to identify the determinants of platform work participation. However, the motivation for using these methods instead of more traditional statistical approaches could be elaborated.
Many of the predictors identified in the analysis (e.g., age, employment status, income conditions) could potentially be examined using conventional econometric models such as logistic regression.
Therefore, the authors should clarify the advantages machine learning provides in this context; how the use of explainable AI contributes to interpreting the results in a social science framework and why the authors did not use traditional statistical models such as logit or probit models..
A clearer discussion of the methodological rationale would strengthen the paper.

RESPONSE: Thank you for this valuable comment. To address this point, we have included logistic regression as a baseline model in the revised manuscript (see Appendix B, Table 4, 5 and 6 - red rows), reporting its hyperparameters and performance metrics. The results show that logistic regression consistently underperforms compared to the others models across all evaluation metrics, suggesting that linear specifications are not sufficient to capture the complexity of platform work participation.
We stress that our use of machine learning is motivated by two main factors. First, it enables a more data-driven approach, reducing ex-ante modelling assumptions and researcher discretion. Second, the richness of the INAPP PLUS data require flexible methods capable of capturing non-linear relationships and interactions across multiple socio-economic dimensions, aiming to achieve a more refined characterisation of the heterogeneous phenomenon of platform work.
Finally, the integration of explainable AI (XAI) ensures interpretability of the ML models, allowing us to extract meaningful insights on the key determinants of platform work in a way that remains consistent with social science analysis.

3. Transparency of the empirical methodology
The description of the empirical procedure would benefit from greater detail to ensure replicability.
In particular, the manuscript should provide clearer information on:
• the definition of the dependent variable (platform work);
• the construction and selection of explanatory variables;
• how missing data were treated;
• whether any feature selection procedures were applied;
• how the dataset was divided into training and test samples;
• the evaluation metrics used to assess model performance.
Providing these details would greatly improve the transparency of the analysis.

RESPONSE: We integrated all missing information in the "Materials and Methods" section, as indicated in red in the text.

4. Interpretation of the results
At several points the manuscript appears to interpret the results in causal terms, although the methodology identifies predictive relationships rather than causal effects.
The authors should therefore adopt a more cautious interpretation and clearly distinguish between:

• predictive associations identified by the models, and
• causal explanations for participation in platform work.
Clarifying this distinction would improve the analytical rigor of the paper.

RESPONSE: Thank you for this important observation. We have: (1) added a methodological disclaimer in the "Explainable AI models" section clarifying that SHAP values do not imply causal relationships, and (2) revised the "Results" and "Discussion" section to replace causal language with strictly correlational phrasing, trying not to overload the reader with repetitions of terminology. All changes are highlighted in red.

5. Role of the pandemic period?
The use of data from both 2018 and 2021 provides an opportunity to capture changes in platform work associated with the COVID-19 pandemic. However, this aspect could be explored in more depth.
Was the profile of platform workers changed between the two years? Has the pandemic affected the expansion of digital labour platforms. A more explicit discussion of these dynamics would enrich the analysis.

RESPONSE: Thank you for this suggestion. Unfortunately, due to insufficient data, we could only train a year-specific model for the 2021 subsample and not for 2018, which limited the depth of the temporal comparison. To address this, we have clarified in the text that our analytical strategy for comparing 2018 and 2021 involved including year as a binary feature in a pooled model and performing a SHAP cohort analysis. We have also softened all causal framing throughout and added references to the existing literature where appropriate.

Other comments

1) **Terminology**
The manuscript occasionally uses terms such as platform work, gig work, and digital labour platforms interchangeably. It would be helpful to provide clearer definitions and maintain consistent terminology throughout the paper.

RESPONSE: We thank the Reviewer for raising this point. We have standardized the terminology and clarified from the outset what we mean by 'platform work'.

2) Structure of the methodology section
The methodology section could benefit from a clearer structure, for example with subsections such as:
• Data
• Variables
• Machine learning models
• Explainable AI methods
• Model validation
This would improve readability.

RESPONSE: We have made minor adjustments to the methodology section to align with the structure recommended by the reviewer, noting that it already matched to most of the prior structure. A cross-reference to Table 3 in the appendix has been included, which lists all final variables and their corresponding values. Information regarding model validation can be found within the sections discussing ML and XAI methods.

3) Discussion section

The discussion could be strengthened by linking the results more explicitly to broader debates on labour market segmentation, precarious employment, and digitalisation.

RESPONSE: We thank the Reviewer for this suggestion. We have reframed the discussion section to more explicitly connect the results to the introduction, particularly with regard to labour market segmentation.
