# Supplementary material for: Platform workers not by chance: Exploring the digital labour markets in Italy with machine learning and explainable AI
Source: PLoS One. 2026 Jun 30;21(6):e0340237. doi: 10.1371/journal.pone.0340237 (PMC13318031; doi:10.1371/journal.pone.0340237)
Supplement: Attachment — Submitted filename: plos one review on platform work .docx [file pone.0340237.s003.docx]

Hello,

Thank you for giving me the opportunity to review this paper. This manuscript addresses an important and timely topic: digital platform work, using high-quality national survey data and advanced machine learning (ML) and explainable AI (XAI) techniques. The empirical material is rich, and the combination of labour sociology with computational methods is a clear strength. However, I have some observations, if these are taken care by authors, the manuscript might be substantially improved and suitable for publications. With targeted revisions, this manuscript can be substantially improved and positioned as a rigorous, transparent, and replicable contribution.

**Overall observations**

(1) The manuscript relies on a broad and somewhat implicit definition of platform work. While this aligns with the PLUS survey structure, the analytical consequences of this definition are not sufficiently discussed. The distinction between primary vs. secondary platform income is central to your argument, yet it is not clearly operationalised upfront. Different forms of platform work (location-based vs web-based; task-based vs continuous) are mentioned theoretically but not analytically differentiated. It does matter because without conceptual tightening, readers may question whether the observed heterogeneity reflects: real labour-market stratification, or definitional breadth and measurement noise.

Therefore, authors can add a short subsection in Materials and Methods explicitly clarifying how PLUS defines platform work; what types of activities are included/excluded; how dependence on platform income is measured. Explicitly acknowledge that heterogeneity may partly stem from definitional aggregation and explain why this does not invalidate the findings.

(2) While ML and XAI are carefully implemented, the manuscript does not sufficiently justify why ML is necessary for the research questions posed. Many of the conclusions (e.g., vulnerability, age gradients, regional divides) could plausibly emerge from logistic regression or multinomial models. The paper emphasises “non-linearities” and “complex interactions” but rarely demonstrates what ML reveals that standard models would miss. It is important to clear because readers often require a clear methodological added value, not just technical sophistication. So , authors can add a benchmark comparison (even in an appendix): e.g., logistic regression vs CatBoost/XGBoost for RQ2 and RQ3., and explicitly state: which relationships are non-linear; which interactions would be difficult to pre-specify in traditional models.
(3) (3)In several places, SHAP-based importance rankings are discussed in language that implies causality or behavioural motivation (e.g., “factors lead jobseekers to turn to platform work”).

Authors can consider the overestimation wording; for example, replace “leads to,” “drives,” “is strongly associated with,” “is predictive of,” or “contributes to the model’s classification.” Also can add a clear methodological disclaimer early in the Results section explaining: SHAP ≠ causal inference.

(4)The manuscript frequently references COVID-19 as an amplifier of platform work dynamics, but: the analytical strategy for comparing 2018 vs 2021 is not fully explicit; it remains unclear whether observed differences reflect pandemic effects or compositional changes.

Could you please clarify whether: models were pooled with year dummies, or estimated separately by year. Include at least one interaction or stratified analysis by year to support pandemic-specific claims. Alternatively, soften claims and frame COVID-19 as a contextual backdrop rather than an identified causal shock.

(5) The PLUS survey is strong, but platform work is notoriously under-reported in telephone surveys. So you can add a dedicated Limitations subsection addressing under-coverage of informal or migrant platform workers; recall bias and misclassification; survivorship bias (those who exited platform work are invisible); implications of survey weights in ML contexts. This will substantially strengthen the paper’s credibility.In addition, could authors work on the following issues in terms of the sections of manuscript:

***Section-specific comments***

**Abstract**

Slightly reduce normative language (“extractive tendencies”). Clarify that findings are associational and predictive.

**Introduction**

Authors did an excellent synthesis of labour sociology and platform economy literature with strong contextualisation of Italy. Yet explicitly link each theoretical strand to a variable or modelling choice. RQ3 implicitly suggests causal pathways (“which factors lead jobseekers…”). Rephrase RQ3 to emphasise probabilistic association, not causation.

***Methods***

Preprocessing steps are dense and hard to follow for non-ML readers. Add a schematic workflow figure. Move some technical detail to an appendix.

**Results**

Some repetition across RQ1 and RQ2. Cluster labels (e.g., “accumulationists,” “sheltered youth”) are evocative but subjective. However, Justify cluster naming more explicitly. Add a concise summary table of cluster characteristics.

**Discussion and conclusions**

Normative tone occasionally exceeds evidence. Policy implications could be more concrete. Authors could separate empirical conclusions from normative reflections. Add 2–3 clearly specified policy implications (e.g., social protection design, labour statistics).
