# Supplementary material for: Platform workers not by chance: Exploring the digital labour markets in Italy with machine learning and explainable AI
Source: PLoS One. 2026 Jun 30;21(6):e0340237. doi: 10.1371/journal.pone.0340237 (PMC13318031; doi:10.1371/journal.pone.0340237)
Supplement: Peer review history

# Author comment: Author response to Decision Letter 0

**DOI:** 10.1371/journal.pone.0340237.r001

11 Dec 2025

Attachment Submitted filename: Response to reviewers.pdf

# Peer review: Decision Letter 0

**DOI:** 10.1371/journal.pone.0340237.r002

**Authors:** Vincenzo Auriemma

15 Mar 2026

PONE-D-25-64501

Platform workers not by chance: exploring the digital labour markets in Italy with machine learning and explainable AI

PLOS One

Dear Dr. Punzi,

Thank you for submitting your manuscript to PLOS ONE. After careful consideration, we feel that it has merit but does not fully meet PLOS ONE’s publication criteria as it currently stands. Therefore, we invite you to submit a revised version of the manuscript that addresses the points raised during the review process.

The review process is now complete; please follow the instructions provided by the two reviewers

Please submit your revised manuscript by Apr 29 2026 11:59PM. If you will need more time than this to complete your revisions, please reply to this message or contact the journal office at plosone@plos.org. When you're ready to submit your revision, log on to https://www.editorialmanager.com/pone/ and select the 'Submissions Needing Revision' folder to locate your manuscript file.

Please include the following items when submitting your revised manuscript:

- A letter that responds to each point raised by the academic editor and reviewer(s). You should upload this letter as a separate file labeled 'Response to Reviewers'.
- A marked-up copy of your manuscript that highlights changes made to the original version. You should upload this as a separate file labeled 'Revised Manuscript with Track Changes'.
- An unmarked version of your revised paper without tracked changes. You should upload this as a separate file labeled 'Manuscript'.

If you would like to make changes to your financial disclosure, please include your updated statement in your cover letter. Guidelines for resubmitting your figure files are available below the reviewer comments at the end of this letter.

If applicable, we recommend that you deposit your laboratory protocols in protocols.io to enhance the reproducibility of your results. Protocols.io assigns your protocol its own identifier (DOI) so that it can be cited independently in the future. For instructions see: https://journals.plos.org/plosone/s/submission-guidelines#loc-laboratory-protocols. Additionally, PLOS ONE offers an option for publishing peer-reviewed Lab Protocol articles, which describe protocols hosted on protocols.io. Read more information on sharing protocols at https://plos.org/protocols?utm_medium=editorial-email&utm_source=authorletters&utm_campaign=protocols.

We look forward to receiving your revised manuscript.

Kind regards,

Vincenzo Auriemma

Academic Editor

PLOS One

Journal Requirements:

When submitting your revision, we need you to address these additional requirements.

1. Please ensure that your manuscript meets PLOS ONE's style requirements, including those for file naming. The PLOS ONE style templates can be found at

https://journals.plos.org/plosone/s/file?id=wjVg/PLOSOne_formatting_sample_main_body.pdf and https://journals.plos.org/plosone/s/file?id=ba62/PLOSOne_formatting_sample_title_authors_affiliations.pdf

2. Please note that PLOS One has specific guidelines on code sharing for submissions in which author-generated code underpins the findings in the manuscript. In these cases, we expect all author-generated code to be made available without restrictions upon publication of the work. Please review our guidelines at https://journals.plos.org/plosone/s/materials-and-software-sharing#loc-sharing-code and ensure that your code is shared in a way that follows best practice and facilitates reproducibility and reuse.

3. We note that you have indicated that there are restrictions to data sharing for this study. For studies involving human research participant data or other sensitive data, we encourage authors to share de-identified or anonymized data. However, when data cannot be publicly shared for ethical reasons, we allow authors to make their data sets available upon request. For information on unacceptable data access restrictions, please see http://journals.plos.org/plosone/s/data-availability#loc-unacceptable-data-access-restrictions.

Before we proceed with your manuscript, please address the following prompts:

a) If there are ethical or legal restrictions on sharing a de-identified data set, please explain them in detail (e.g., data contain potentially identifying or sensitive patient information, data are owned by a third-party organization, etc.) and who has imposed them (e.g., a Research Ethics Committee or Institutional Review Board, etc.). Please also provide contact information for a data access committee, ethics committee, or other institutional body to which data requests may be sent.

b) If there are no restrictions, please upload the minimal anonymized data set necessary to replicate your study findings to a stable, public repository and provide us with the relevant URLs, DOIs, or accession numbers. Please see http://www.bmj.com/content/340/bmj.c181.long for guidelines on how to de-identify and prepare clinical data for publication. For a list of recommended repositories, please see https://journals.plos.org/plosone/s/recommended-repositories. You also have the option of uploading the data as Supporting Information files, but we would recommend depositing data directly to a data repository if possible.

Please update your Data Availability statement in the submission form accordingly.

4. We note that the grant information you provided in the ‘Funding Information’ and ‘Financial Disclosure’ sections do not match.

When you resubmit, please ensure that you provide the correct grant numbers for the awards you received for your study in the ‘Funding Information’ section.

5. Please upload a copy of Figure 12, to which you refer in your text on page 11. If the figure is no longer to be included as part of the submission please remove all reference to it within the text.

6. Please include captions for your Supporting Information files at the end of your manuscript, and update any in-text citations to match accordingly. Please see our Supporting Information guidelines for more information: http://journals.plos.org/plosone/s/supporting-information.

7. If the reviewer comments include a recommendation to cite specific previously published works, please review and evaluate these publications to determine whether they are relevant and should be cited. There is no requirement to cite these works unless the editor has indicated otherwise.

[Note: HTML markup is below. Please do not edit.]

Reviewers' comments:

Reviewer's Responses to Questions

**Comments to the Author**

1. Is the manuscript technically sound, and do the data support the conclusions?

The manuscript must describe a technically sound piece of scientific research with data that supports the conclusions. Experiments must have been conducted rigorously, with appropriate controls, replication, and sample sizes. The conclusions must be drawn appropriately based on the data presented.

Reviewer #1: Yes

Reviewer #2: Yes

**********

2. Has the statistical analysis been performed appropriately and rigorously?

Reviewer #1: Yes

Reviewer #2: Yes

**********

3. Have the authors made all data underlying the findings in their manuscript fully available?

The PLOS Data policy requires authors to make all data underlying the findings described in their manuscript fully available without restriction, with rare exception (please refer to the Data Availability Statement in the manuscript PDF file). The data should be provided as part of the manuscript or its supporting information, or deposited to a public repository. For example, in addition to summary statistics, the data points behind means, medians and variance measures should be available. If there are restrictions on publicly sharing data—e.g. participant privacy or use of data from a third party—those must be specified.

Reviewer #1: Yes

Reviewer #2: Yes

**********

4. Is the manuscript presented in an intelligible fashion and written in standard English?

PLOS ONE does not copyedit accepted manuscripts, so the language in submitted articles must be clear, correct, and unambiguous. Any typographical or grammatical errors should be corrected at revision, so please note any specific errors here.

Reviewer #1: Yes

Reviewer #2: Yes

**********

5. Review Comments to the Author

Please use the space provided to explain your answers to the questions above. You may also include additional comments for the author, including concerns about dual publication, research ethics, or publication ethics. (Please upload your review as an attachment if it exceeds 20,000 characters)

Reviewer #1: This paper is well written.However, this manuscript require major revision for publication. I have provided some technical or methodolgocal commets in the attached file. If authors can address the issues raised, it will be an excellent work and ready for publication.

Reviewer #2: The manuscript “Platform workers not by chance: exploring the digital labour markets in Italy with machine learning and explainable AI” investigates the determinants of participation in platform work in Italy using survey data from 2018 and 2021. The authors apply machine learning techniques combined with explainable AI tools to identify the main predictors of participation in digital labour platforms.

The topic is timely and relevant. Platform work has become an increasingly important component of contemporary labour markets, and empirical evidence for Italy remains relatively limited. The attempt to apply machine learning and explainable AI methods to labour market analysis is potentially valuable, especially for identifying complex patterns in large datasets.

Overall, the manuscript has the potential to make a useful contribution. However, several aspects of the paper need clarification and improvement, especially with reference to the positioning of the contribution within the existing literature, the justification for the use of machine learning methods, and the transparency of the empirical methodology.

For these reasons, I recommend major revisions before the manuscript can be considered for publication.

Major comments

1. Positioning within the literature

While the manuscript reviews a number of studies on platform work, the specific contribution of the paper relative to the existing literature could be articulated more clearly.

In particular, it would be useful to clarify the empirical or methodological gap the study aims to fill and how the findings extend or challenge previous research on platform work and labour market segmentation;

The paper suggests that platform work is not predominantly a youth phenomenon but is instead associated with economically vulnerable workers. This is an interesting claim, but it should be discussed more systematically in relation to the existing literature on labour market dualism, precarious employment, and gig work.

2. Justification for the use of machine learning

The manuscript relies on machine learning techniques combined with explainable AI tools to identify the determinants of platform work participation. However, the motivation for using these methods instead of more traditional statistical approaches could be elaborated.

Many of the predictors identified in the analysis (e.g., age, employment status, income conditions) could potentially be examined using conventional econometric models such as logistic regression.

Therefore, the authors should clarify the advantages machine learning provides in this context; how the use of explainable AI contributes to interpreting the results in a social science framework and why the authors did not use traditional statistical models such as logit or probit models..

A clearer discussion of the methodological rationale would strengthen the paper.

3. Transparency of the empirical methodology

The description of the empirical procedure would benefit from greater detail to ensure replicability.

In particular, the manuscript should provide clearer information on:

• the definition of the dependent variable (platform work);

• the construction and selection of explanatory variables;

• how missing data were treated;

• whether any feature selection procedures were applied;

• how the dataset was divided into training and test samples;

• the evaluation metrics used to assess model performance.

Providing these details would greatly improve the transparency of the analysis.

4. Interpretation of the results

At several points the manuscript appears to interpret the results in causal terms, although the methodology identifies predictive relationships rather than causal effects.

The authors should therefore adopt a more cautious interpretation and clearly distinguish between:

• predictive associations identified by the models, and

• causal explanations for participation in platform work.

Clarifying this distinction would improve the analytical rigor of the paper.

5. Role of the pandemic period?

The use of data from both 2018 and 2021 provides an opportunity to capture changes in platform work associated with the COVID-19 pandemic. However, this aspect could be explored in more depth.

Was the profile of platform workers changed between the two years? Has the pandemic affected the expansion of digital labour platforms. A more explicit discussion of these dynamics would enrich the analysis.

Terminology

The manuscript occasionally uses terms such as platform work, gig work, and digital labour platforms interchangeably. It would be helpful to provide clearer definitions and maintain consistent terminology throughout the paper.

Structure of the methodology section

The methodology section could benefit from a clearer structure, for example with subsections such as:

• Data

• Variables

• Machine learning models

• Explainable AI methods

• Model validation

This would improve readability.

Discussion section

The discussion could be strengthened by linking the results more explicitly to broader debates on labour market segmentation, precarious employment, and digitalisation.

**********

6. PLOS authors have the option to publish the peer review history of their article (what does this mean?). If published, this will include your full peer review and any attached files.

If you choose “no”, your identity will remain anonymous but your review may still be made public.

**Do you want your identity to be public for this peer review?**  For information about this choice, including consent withdrawal, please see our Privacy Policy.

Reviewer #1: No

Reviewer #2: No

**********

[NOTE: If reviewer comments were submitted as an attachment file, they will be attached to this email and accessible via the submission site. Please log into your account, locate the manuscript record, and check for the action link "View Attachments". If this link does not appear, there are no attachment files.]

To ensure your figures meet our technical requirements, please review our figure guidelines: https://journals.plos.org/plosone/s/figures

You may also use PLOS’s free figure tool, NAAS, to help you prepare publication quality figures: https://journals.plos.org/plosone/s/figures#loc-tools-for-figure-preparation.

NAAS will assess whether your figures meet our technical requirements by comparing each figure against our figure specifications.

Attachment Submitted filename: plos one review on platform work .docx

# Author comment: Author response to Decision Letter 1

**DOI:** 10.1371/journal.pone.0340237.r003

4 May 2026

Dear Editor,

We sincerely thank you for the opportunity to revise and resubmit our manuscript.

We have carefully addressed all the valuable suggestions provided by the reviewers, which we believe have substantially improved the clarity and overall effectiveness of our contribution. In particular, we have strengthened both the methodology and the interpretation of the results, and we have better framed our analysis in relation to the existing literature on platform and non-standard work.

In the following, we provide detailed responses to each of the reviewers’ comments. All revisions and additions are highlighted in red in the main text.

Sincerely yours,

Reviewer #1:

Overall observations

(1) The manuscript relies on a broad and somewhat implicit definition of platform work. While this aligns with the PLUS survey structure, the analytical consequences of this definition are not sufficiently discussed. The distinction between primary vs. secondary platform income is central to your argument, yet it is not clearly operationalised upfront. Different forms of platform work (location-based vs web-based; task-based vs continuous) are mentioned theoretically but not analytically differentiated. It does matter because without conceptual tightening, readers may question whether the observed heterogeneity reflects: real labour-market stratification, or definitional breadth and measurement noise.

Therefore, authors can add a short subsection in Materials and Methods explicitly clarifying how PLUS defines platform work; what types of activities are included/excluded; how dependence on platform income is measured. Explicitly acknowledge that heterogeneity may partly stem from definitional aggregation and explain why this does not invalidate the findings.

RESPONSE: Thank you for this thoughtful comment. In response, we have made the following additions to the “Data” section. First, we have explicitly added the definition of platform work as operationalised by the PLUS survey, including the exact wording of the screening question (same for 2018 and 2021). Second, we now clearly state that the limited sample size prevents differentiated analyses across platform task types (e.g., delivery vs. online tasks) or by primary versus secondary platform income. We explain that this aggregation does not invalidate our findings, as different task types share common structural features, such as precarious conditions, economic vulnerability, and limited protections. Third, while being transparent about these limitations, we have added descriptive statistics on platform tasks and occupational status providing an additional and insightful piece of information concerning the characteristics of platform work in Italy.

(2) While ML and XAI are carefully implemented, the manuscript does not sufficiently justify why ML is necessary for the research questions posed. Many of the conclusions (e.g., vulnerability, age gradients, regional divides) could plausibly emerge from logistic regression or multinomial models. The paper emphasises “non-linearities” and “complex interactions” but rarely demonstrates what ML reveals that standard models would miss. It is important to clear because readers often require a clear methodological added value, not just technical sophistication. So, authors can add a benchmark comparison (even in an appendix): e.g., logistic regression vs CatBoost/XGBoost for RQ2 and RQ3., and explicitly state: which relationships are non-linear and which interactions would be difficult to pre-specify in traditional models.

RESPONSE: We thank the reviewer for this important comment. To clarify the methodological contribution of the paper, we have now included a benchmark comparison with logistic regression models for both RQ2 and RQ3 (reported in Appendix B, Table 4 and 5 - red rows). The results show that logistic regression consistently underperforms relative to the selected ML models (CatBoost and XGBoost) across all evaluation metrics, suggesting that the relationships underlying platform work participation are not fully captured by linear specifications. This supports our initial motivation for employing ML methods, namely their ability to flexibly capture non-linear patterns and higher-order interactions without requiring strong a priori assumptions. We have revised the methodology section to make this justification more explicit and better highlight our methodological contribution.

(3) In several places, SHAP-based importance rankings are discussed in language that implies causality or behavioural motivation (e.g., “factors lead jobseekers to turn to platform work”).

Authors can consider the overestimation wording; for example, replace “leads to,” “drives,” “is strongly associated with,” “is predictive of,” or “contributes to the model’s classification.” Also can add a clear methodological disclaimer early in the Results section explaining: SHAP ≠ causal inference.

RESPONSE: Thank you for this important observation. We have: (1) added a methodological disclaimer in the “Explainable AI models” section clarifying that SHAP values do not imply causal relationships, and (2) revised the “Results” and “Discussion” section to replace causal language with strictly correlational phrasing, trying not to overload the reader with repetitions of terminology. All changes are highlighted in red.

(4) The manuscript frequently references COVID-19 as an amplifier of platform work dynamics, but: the analytical strategy for comparing 2018 vs 2021 is not fully explicit; it remains unclear whether observed differences reflect pandemic effects or compositional changes.

Could you please clarify whether: models were pooled with year dummies, or estimated separately by year. Include at least one interaction or stratified analysis by year to support pandemic-specific claims. Alternatively, soften claims and frame COVID-19 as a contextual backdrop rather than an identified causal shock.

RESPONSE: Thank you for this observation. We have clarified in the text the analytical strategy for comparing 2018 vs 2021: as limited sample size prevented separate year-specific models, we included year as a binary feature in a pooled model and performed SHAP cohort analysis accordingly. We have also softened all causal framing, adding references to the existing literature where appropriate.

(5) The PLUS survey is strong, but platform work is notoriously under-reported in telephone surveys. So you can add a dedicated Limitations subsection addressing under-coverage of informal or migrant platform workers; recall bias and misclassification; survivorship bias (those who exited platform work are invisible); implications of survey weights in ML contexts. This will substantially strengthen the paper’s credibility.

RESPONSE: Thank you for these constructive suggestions. Please note that most of the concerns raised were already addressed in the section now renamed "Limitations" (formerly "Conclusion"). In response to your comment, we have additionally: (1) added a specific reference to survivorship bias; (2) explicitly mentioned informal workers alongside migrants when discussing under-coverage; (3) clarified that misclassification measures are reported in the Appendix. Also note that survey weights were applied in all classification models, as specified in the Methods section.

Section-specific comments

1) Abstract

Slightly reduce normative language (“extractive tendencies”). Clarify that findings are associational and predictive.

RESPONSE: We thank the Reviewer for raising this point. We have reduced the use of normative language and clarified the non-causal nature of both the analysis and the results.

2) Introduction

Authors did an excellent synthesis of labour sociology and platform economy literature with strong contextualisation of Italy. Yet explicitly link each theoretical strand to a variable or modelling choice. RQ3 implicitly suggests causal pathways (“which factors lead jobseekers…”). Rephrase RQ3 to emphasise probabilistic association, not causation.

RESPONSE: We thank the Reviewer for raising this point. In response, we have added a new paragraph in the subsection ‘Scope and Structure of the Study’ that explicitly links the groups of variables used in the analysis to the relevant literature. Moreover, we have rephrased RQ3 to remove causal language.

3) Methods

Preprocessing steps are dense and hard to follow for non-ML readers. Add a schematic workflow figure. Move some technical detail to an appendix.

RESPONSE: Thank you for this helpful suggestion. In response, we have added a schematic workflow figure (now Figure 1) that visually summarises the main steps of our explainable machine learning pipeline, making the overall methodology more accessible to non-ML readers. However, we have retained the detailed preprocessing descriptions in the main text, as another reviewer specifically requested more technical detail.

4) Results

Some repetition across RQ1 and RQ2. Cluster labels (e.g., “accumulationists,” “sheltered youth”) are evocative but subjective. However, Justify cluster naming more explicitly. Add a concise summary table of cluster characteristics.

RESPONSE: We thank the reviewer for this comment. To enhance the reader's understanding of the proposed terminology for the clusters, we have included a concise summary table of cluster characteristics (Table 2, red background), as well as a comprehensive set of descriptive statistics, encompassing modes, means, and standard deviations for all variables by cluster in Table 7 (red background) of the Appendix.

5) Discussion and conclusions

Normative tone occasionally exceeds evidence. Policy implications could be more concrete. Authors could separate empirical conclusions from normative reflections. Add 2–3 clearly specified policy implications (e.g., social protection design, labour statistics).

RESPONSE: We thank the reviewer for raising this point. We have revised the conclusions including policy implications and removing normative tone.

Reviewer #2:

Major comments

1. Positioning within the literature

While the manuscript reviews a number of studies on platform work, the specific contribution of the paper relative to the existing literature could be articulated more clearly.

In particular, it would be useful to clarify the empirical or methodological gap the study aims to fill and how the findings extend or challenge previous research on platform work and labour market segmentation;

The paper suggests that platform work is not predominantly a youth phenomenon but is instead associated with economically vulnerable workers. This is an interesting claim, but it should be discussed more systematically in relation to the existing literature on labour market dualism, precarious employment, and gig work.

RESPONSE: We thank the reviewer for raising this point. We have rewritten the introduction to explicitly refer to contributions on labour market dualism in order to better frame our analysis.

2. Justification for the use of machine learning

The manuscript relies on machine learning techniques combined with explainable AI tools to identify the determinants of platform work participation. However, the motivation for using these methods instead of more traditional statistical approaches could be elaborated.

Many of the predictors identified in the analysis (e.g., age, employment status, income conditions) could potentially be examined using conventional econometric models such as logistic regression.

Therefore, the authors should clarify the advantages machine learning provides in this context; how the use of explainable AI contributes to interpreting the results in a social science framework and why the authors did not use traditional statistical models such as logit or probit models..

A clearer discussion of the methodological rationale would strengthen the paper.

RESPONSE: Thank you for this valuable comment. To address this point, we have included logistic regression as a baseline model in the revised manuscript (see Appendix B, Table 4, 5 and 6 - red rows), reporting its hyperparameters and performance metrics. The results show that logistic regression consistently underperforms compared to the others models across all evaluation metrics, suggesting that linear specifications are not sufficient to capture the complexity of platform work participation.

We stress that our use of machine learning is motivated by two main factors. First, it enables a more data-driven approach, reducing ex-ante modelling assumptions and researcher discretion. Second, the richness of the INAPP PLUS data require flexible methods capable of capturing non-linear relationships and interactions across multiple socio-economic dimensions, aiming to achieve a more refined characterisation of the heterogeneous phenomenon of platform work.

Finally, the integration of explainable AI (XAI) ensures interpretability of the ML models, allowing us to extract meaningful insights on the key determinants of platform work in a way that remains consistent with social science analysis.

3. Transparency of the empirical methodology

The description of the empirical procedure would benefit from greater detail to ensure replicability.

In particular, the manuscript should provide clearer information on:

• the definition of the dependent variable (platform work);

• the construction and selection of explanatory variables;

• how missing data were treated;

• whether any feature selection procedures were applied;

• how the dataset was divided into training and test samples;

• the evaluation metrics used to assess model performance.

Providing these details would greatly improve the transparency of the analysis.

RESPONSE: We integrated all missing information in the "Materials and Methods" section, as indicated in red in the text.

4. Interpretation of the results

At several points the manuscript appears to interpret the results in causal terms, although the methodology identifies predictive relationships rather than causal effects.

The authors should therefore adopt a more cautious interpretation and clearly distinguish between:

• predictive associations identified by the models, and

• causal explanations for participation in platform work.

Clarifying this distinction would improve the analytical rigor of the paper.

RESPONSE: Thank you for this important observation. We have: (1) added a methodological disclaimer in the “Explainable AI models” section clarifying that SHAP values do not imply causal relationships, and (2) revised the “Results” and “Discussion” section to replace causal language with strictly correlational phrasing, trying not to overload the reader with repetitions of terminology. All changes are highlighted in red.

5. Role of the pandemic period?

The use of data from both 2018 and 2021 provides an opportunity to capture changes in platform work associated with the COVID-19 pandemic. However, this aspect could be explored in more depth.

Was the profile of platform workers changed between the two years? Has the pandemic affected the expansion of digital labour platforms. A more explicit discussion of these dynamics would enrich the analysis.

RESPONSE: Thank you for this suggestion. Unfortunately, due to insufficient data, we could only train a year-specific model for the 2021 subsample and not for 2018, which limited the depth of the temporal comparison. To address this, we have clarified in the text that our analytical strategy for comparing 2018 and 2021 involved including year as a binary feature in a pooled model and performing a SHAP cohort analysis. We have also softened all causal framing throughout and added references to the existing literature where appropriate.

Other comments

1) Terminology

The manuscript occasionally uses terms such as platform work, gig work, and digital labour platforms interchangeably. It would be helpful to provide clearer definitions and maintain consistent terminology throughout the paper.

RESPONSE: We thank the Reviewer for raising this point. We have standardized the terminology and clarified from the outset what we mean by ‘platform work’.

2) Structure of

Attachment Submitted filename: Response to reviewers_v2.pdf

# Editor report: Decision Letter 1

**DOI:** 10.1371/journal.pone.0340237.r004

**Authors:** Vincenzo Auriemma

31 May 2026

Platform workers not by chance: exploring the digital labour markets in Italy with machine learning and explainable AI

PONE-D-25-64501R1

Dear Dr. Punzi,

We’re pleased to inform you that your manuscript has been judged scientifically suitable for publication and will be formally accepted for publication once it meets all outstanding technical requirements.

Within one week, you’ll receive an e-mail detailing the required amendments. When these have been addressed, you’ll receive a formal acceptance letter and your manuscript will be scheduled for publication.

An invoice will be generated when your article is formally accepted. Please note, if your institution has a publishing partnership with PLOS and your article meets the relevant criteria, all or part of your publication costs will be covered. Please make sure your user information is up-to-date by logging into Editorial Manager at Editorial Manager® and clicking the ‘Update My Information' link at the top of the page. For questions related to billing, please contact billing support.

If your institution or institutions have a press office, please notify them about your upcoming paper to help maximize its impact. If they’ll be preparing press materials, please inform our press team as soon as possible -- no later than 48 hours after receiving the formal acceptance. Your manuscript will remain under strict press embargo until 2 pm Eastern Time on the date of publication. For more information, please contact onepress@plos.org.

Kind regards,

Vincenzo Auriemma

Academic Editor

PLOS One

Additional Editor Comments (optional):

Reviewers' comments:

# Editor report: Acceptance letter

**DOI:** 10.1371/journal.pone.0340237.r005

**Authors:** Vincenzo Auriemma

PONE-D-25-64501R1

PLOS One

Dear Dr. Punzi,

I'm pleased to inform you that your manuscript has been deemed suitable for publication in PLOS One. Congratulations! Your manuscript is now being handed over to our production team.

At this stage, our production department will prepare your paper for publication. This includes ensuring the following:

* All references, tables, and figures are properly cited

* All relevant supporting information is included in the manuscript submission,

* There are no issues that prevent the paper from being properly typeset

You will receive further instructions from the production team, including instructions on how to review your proof when it is ready. Please keep in mind that we are working through a large volume of accepted articles, so please give us a few days to review your paper and let you know the next and final steps.

Lastly, if your institution or institutions have a press office, please let them know about your upcoming paper now to help maximize its impact. If they'll be preparing press materials, please inform our press team within the next 48 hours. Your manuscript will remain under strict press embargo until 2 pm Eastern Time on the date of publication. For more information, please contact onepress@plos.org.

You will receive an invoice from PLOS for your publication fee after your manuscript has reached the completed accept phase. If you receive an email requesting payment before acceptance or for any other service, this may be a phishing scheme. Learn how to identify phishing emails and protect your accounts at https://explore.plos.org/phishing.

If we can help with anything else, please email us at customercare@plos.org.

Thank you for submitting your work to PLOS ONE and supporting open access.

Kind regards,

PLOS ONE Editorial Office Staff

on behalf of

Dr. Vincenzo Auriemma

Academic Editor

PLOS One
